# Treatments and Outcomes Among Patients with Sydenham Chorea: A Meta-Analysis

**DOI:** 10.1001/jamanetworkopen.2024.6792

**Published:** 2024-04-16

**Authors:** Michael Eyre, Terrence Thomas, Emanuela Ferrarin, Sonia Khamis, Sameer M. Zuberi, Adrian Sie, Tamsin Newlove-Delgado, Michael Morton, Erika Molteni, Russell C. Dale, Ming Lim, Margherita Nosadini

**Affiliations:** 1 School of Biomedical Engineering and Imaging Sciences, King’s College London, London, United Kingdom; 2 Children’s Neurosciences, Evelina London Children’s Hospital at Guy’s and St Thomas’ NHS Foundation Trust, London, United Kingdom; 3 Department of Paediatrics, Neurology Service, KK Women’s and Children’s Hospital, Singapore; 4 Centro di Riferimento Oncologico di Aviano IRCCS, Aviano, Italy; 5 Children’s Neurosciences, Evelina London Children’s Hospital at Guy’s and St Thomas’ NHS Foundation Trust, London, United Kingdom; 6 Paediatric Neurosciences Research Group, Royal Hospital for Children, Glasgow, United Kingdom; 7 Institute of Health and Wellbeing, University of Glasgow, Glasgow, United Kingdom; 8 NHS Lanarkshire, Bothwell, United Kingdom; 9 Children and Young People’s Mental Health (ChYMe) Research Collaboration, University of Exeter Medical School, Exeter, United Kingdom; 10 School of Biomedical Engineering and Imaging Sciences, King’s College London, United Kingdom; 11 Kids Neuroscience Centre, The Children’s Hospital at Westmead, Faculty of Medicine and Health, University of Sydney, Westmead, Australia; 12 Faculty of Life Sciences and Medicine, King’s College London, United Kingdom; 13 Children’s Neurosciences, Evelina London Children’s Hospital at Guy’s and St Thomas’ NHS Foundation Trust, London, United Kingdom; 14 Paediatric Neurology and Neurophysiology Unit, Department of Women’s and Children’s Health, University Hospital of Padova, Padova, Italy; 15 Neuroimmunology Group, Paediatric Research Institute “Città della Speranza,” Padova, Italy

## Abstract

**Importance:**

Sydenham chorea is the most common acquired chorea of childhood worldwide; however, treatment is limited by a lack of high-quality evidence.

**Objectives:**

To evaluate historical changes in the clinical characteristics of Sydenham chorea and identify clinical and treatment factors at disease onset associated with chorea duration, relapsing disease course, and functional outcome.

**Data Sources:**

The systematic search for this meta-analysis was conducted in PubMed, Embase, CINAHL, Cochrane Library, and LILACS databases and registers of clinical trials from inception to November 1, 2022 (search terms: [*Sydenham* OR *Sydenham’s* OR *rheumatic* OR *minor*] AND *chorea*).

**Study Selection:**

Published articles that included patients with a final diagnosis of Sydenham chorea (in selected languages).

**Data Extraction and Synthesis:**

This study followed the Preferred Reporting Items for Systematic Reviews and Meta-analyses (PRISMA) reporting guideline. Individual patient data on clinical characteristics, treatments, chorea duration, relapse, and final outcome were extracted. Data from patients in the modern era (1945 through 2022) were entered into multivariable models and stratified by corticosteroid duration for survival analysis of chorea duration.

**Main Outcomes and Measures:**

The planned study outcomes were chorea duration at onset, monophasic course (absence of relapse after ≥24 months), and functional outcome (poor: modified Rankin Scale score 2-6 or persisting chorea, psychiatric, or behavioral symptoms at final follow-up after ≥6 months; good: modified Rankin Scale score 0-1 and no chorea, psychiatric, or behavioral symptoms at final follow-up).

**Results:**

In total, 1479 patients were included (from 307 articles), 1325 since 1945 (median [IQR] age at onset, 10 [8-13] years; 875 of 1272 female [68.8%]). Immunotherapy was associated with shorter chorea duration (hazard ratio for chorea resolution, 1.51 [95% CI, 1.05-2.19]; *P* = .03). The median chorea duration in patients receiving 1 or more months of corticosteroids was 1.2 months (95% CI, 1.2-2.0) vs 2.8 months (95% CI, 2.0-3.0) for patients receiving none (*P* = .004). Treatment factors associated with monophasic disease course were antibiotics (odds ratio [OR] for relapse, 0.28 [95% CI, 0.09-0.85]; *P* = .02), corticosteroids (OR, 0.32 [95% CI, 0.15-0.67]; *P* = .003), and sodium valproate (OR, 0.33 [95% CI, 0.15-0.71]; *P* = .004). Patients receiving at least 1 month of corticosteroids had significantly lower odds of relapsing course (OR, 0.10 [95% CI, 0.04-0.25]; *P* < .001). No treatment factor was associated with good functional outcome.

**Conclusions and Relevance:**

In this meta-analysis of treatments and outcomes in patients with Sydenham chorea, immunotherapy, in particular corticosteroid treatment, was associated with faster resolution of chorea. Antibiotics, corticosteroids and sodium valproate were associated with a monophasic disease course. This synthesis of retrospective data should support the development of evidence-based treatment guidelines for patients with Sydenham chorea.

## Key Points

**Question:**

Which clinical and treatment factors at onset of Sydenham chorea are associated with chorea duration, relapsing disease course, and functional outcome?

**Findings:**

In this individual patient data meta-analysis of 1479 patients, those receiving at least 1 month of corticosteroids had a median chorea duration of 1.2 months vs 2.8 months for patients receiving none, a significant difference. Patients treated with antibiotics, corticosteroids, or sodium valproate had significantly reduced odds of relapse.

**Meaning:**

These observational data support the use of corticosteroids, antibiotics, and sodium valproate for treatment of Sydenham chorea.

## Teaser

This individual patient data meta-analysis investigates historical changes in the clinical characteristics of Sydenham chorea and identifies clinical and treatment factors at disease onset associated with chorea duration, relapsing disease course, and functional outcome among patients with the disorder.

## Introduction

Sydenham chorea (SC) is an autoimmune neuropsychiatric disorder associated with prior group A streptococcal (GAS) infection.^1^ It is 1 of the major manifestations of acute rheumatic fever (ARF) and remains the most common acquired chorea of childhood worldwide, including some high-income settings.^2,3^ In SC, chorea of the extremities, and, often, chorea of the face, tongue, and trunk are usually accompanied by hypotonia and emotional or behavioral disturbances, most frequently emotional lability.^1^ In a subgroup with severe disease, there is complete loss of tone and voluntary movements (chorea paralytica).^4,5^ Most patients recover fully within 6 to 9 months, but symptoms persist in up to 40% of patients, ^1,6,7,8^ and relapses occur in 16%-42%.^9,10,11,12^ Except for antibiotics, there is limited consensus regarding treatment.^1,13^ Only 3 small randomized clinical trials (RCTs) assessing immunotherapy for SC have been reported,^14,15,16^ and treatment strategies vary widely, with corticosteroid use in recent cohorts ranging from 16% to 75%, and steroid regimens differing even within centers.^17,18,19,20^ Herein we present a comprehensive evidence synthesis of published SC cases with individual patient data (IPD), with the aims of describing the clinical features and management of SC and of identifying associations between early clinical and treatment factors and disease course and outcome.

## Methods

### Literature Search and Data Collection

For this meta-analysis, PubMed, Embase, CINAHL, Cochrane Library, and LILACS (Literatura Latino-Americana e do Caribe em Ciências da Saúde) databases and registers of clinical trials were searched from inception to November 1, 2022 (search terms: [*Sydenham* OR *Sydenham’s* OR *rheumatic* OR *minor*] AND *chorea*). eFigure 1 and eTable 1 in Supplement 1 provide search and article selection details. Cases from articles in selected languages (English, French, Spanish, Portuguese, and Italian) with IPD were included if they included acute or subacute chorea onset and a final diagnosis of SC according to the authors. If not provided, IPD was requested from the authors of articles published since January 1, 2012, reporting at least 10 cases. Individual patient data on demographics, preexisting conditions, symptoms, severity at the first SC episode, ARF manifestations, treatments, and outcomes were collected using a standardized proforma (eMethods 1 in Supplement 1). This study followed the Preferred Reporting Items for Systematic Reviews and Meta-analyses (PRISMA) reporting guideline.

### Study Outcomes

We evaluated 3 main outcomes: chorea duration at the first SC episode, relapsing disease course, and final functional outcome. Relapsing disease course was defined as the occurrence of at least 1 relapse (at any time); monophasic disease course was defined as no relapse after a minimum of 24 months’ follow-up. Poor functional outcome was defined as a modified Rankin Scale (mRS) score of 2 to 6 or persisting chorea or psychiatric or behavioral symptoms at final follow-up 6 or more months after the last SC episode. Good functional outcome was defined as an mRS score of 0 to 1 and no chorea or psychiatric or behavioral symptoms at final follow-up (at any time).

### Statistical Analysis

For historical comparison, patients with disease onset (or if unknown, publication year) before 1945 (when penicillin first became commercially available and 1 year after the first ARF diagnostic criteria were established^21,22^) were compared with patients from the modern era (1945 through 2022) using the χ^2^ or Fisher exact test for nominal data, Mann-Whitney *U* test for continuous or ordinal data, and Kaplan-Meier survival analysis with the log-rank test for chorea duration. To optimize data reliability and relevance, only data since 1945 were included in subsequent analyses. Denominators for descriptive data varied according to data availability. Symptomatic medications were grouped into pharmacological classes, and differences in clinician-reported benefit were tested using pairwise χ^2^ tests with Bonferroni-corrected *P* values. Medication classes given to fewer than 10 patients were not included. To assess the 3 main outcomes, 3 separate multivariable models were applied: a Cox proportional hazards regression model for chorea duration at first episode (including symptomatic medications and immunotherapy as time-varying features) and logistic regression models for relapsing disease course and functional outcome. Missing values for 27 variables (eTable 2 in Supplement 1) underwent hot-deck imputation prior to multivariable modeling (eMethods 2 in Supplement 1).^23,24^ Sensitivity analyses were conducted for year of onset and missingness (eMethods 3 in Supplement 1). In further univariate analyses of the nonimputed data, patients were grouped according to corticosteroid treatment duration at the first episode (none, <1 month, or ≥1 month) to evaluate associations with chorea duration (Kaplan-Meier survival analysis) and relapsing course (Fisher exact tests). Two-tailed *P* < .05 was regarded as significant. Analyses used Python, version 3.10 (Python Software Foundation) with statsmodels, lifelines, and hail packages.

## Results

### Historical Trends

**Figure 1.**
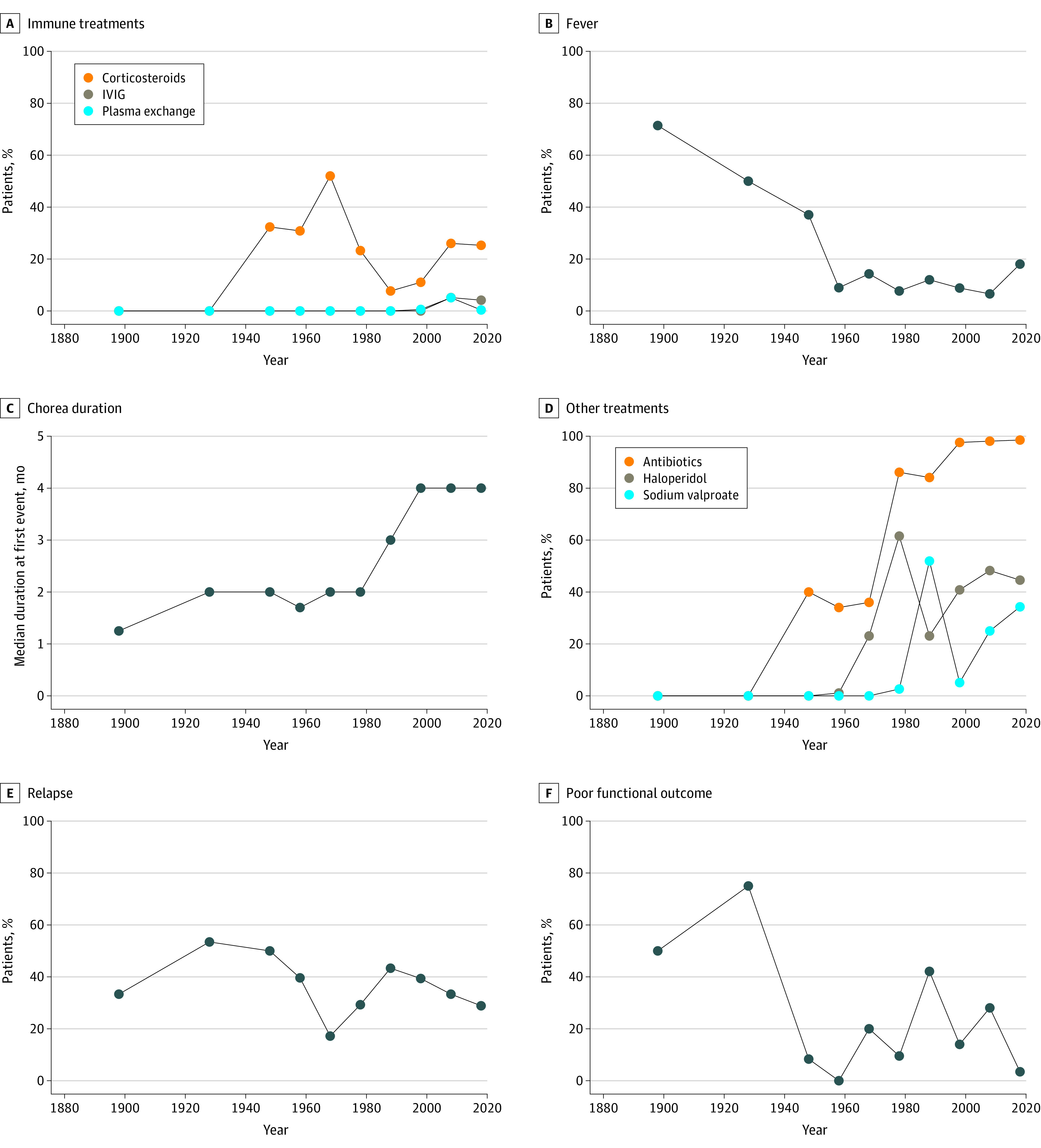
Historical Trends in Sydenham Chorea Plotted points represent patients grouped into 10 epochs: 1883-1912, 1913-1942, and 10-year intervals thereafter. IVIG indicates intravenous immunoglobulin.

We identified 1479 patients with IPD (median [IQR] age at onset, 10 [8-13] years in 1354 patients; 985 of 1426 [69.1%] female and 441 of 1426 [30.9%] male) were identified from 307 articles^25,26,27,28,29,30,31,32,33,34,35,36,37,38,39,40,41,42,43,44,45,46,47,48,49,50,51,52,53,54,55,56,57,58,59,60,61,62,63,64,65,66,67,68,69,70,71,72,73,74,75,76,77,78,79,80,81,82,83,84,85,86,87,88,89,90,91,92,93,94,95,96,97,98,99,100,101,102,103,104,105,106,107,108,109,110,111,112,113,114,115,116,117,118,119,120,121,122,123,124,125,126,127,128,129,130,131,132,133,134,135,136,137,138,139,140,141,142,143,144,145,146,147,148,149,150,151,152,153,154,155,156,157,158,159,160,161,162,163,164,165,166,167,168,169,170,171,172,173,174,175,176,177,178,179,180,181,182,183,184,185,186,187,188,189,190,191,192,193,194,195,196,197,198,199,200,201,202,203,204,205,206,207,208,209,210,211,212,213,214,215,216,217,218,219,220,221,222,223,224,225,226,227,228,229,230,231,232,233,234,235,236,237,238,239,240,241,242,243,244,245,246,247,248,249,250,251,252,253,254,255,256,257,258,259,260,261,262,263,264,265,266,267,268,269,270,271,272,273,274,275,276,277,278,279,280,281,282,283,284,285,286,287,288,289,290,291,292,293,294,295,296,297,298,299,300,301,302,303,304,305,306,307,308,309,310,311,312,313,314,315,316,317,318,319,320,321,322,323,324,325,326,327,328,329,330,331^ (eFigure 1 in Supplement 1). Compared with 1325 patients identified in the modern era (1945 to 2022), 154 patients with onset before 1945 had more frequent fever (11 of 20 [55.0%] vs 66 of 458 [14.4%]) and worse severity (median [IQR] mRS, 4 [3-4] vs 3 [3-4]) during the first SC episode, longer hospitalization (median, 40 [25-62] days vs 21 [10-35] days), more frequent arthritis or arthralgia (48 of 145 patients [33.1%] vs 275 of 1118 patients [24.6%]), shorter chorea duration at first episode (median IQR, 2.0 [1.0-3.0] months vs 3.0 [1.2-6.0] months) and worse long-term outcome (5 of 13 patients [39%] vs 47 of 338 patients [13.9%] with poor functional outcome) (Figure 1 and eTable 3 in Supplement 1).

### Demographics and Background History

**Figure 2.**
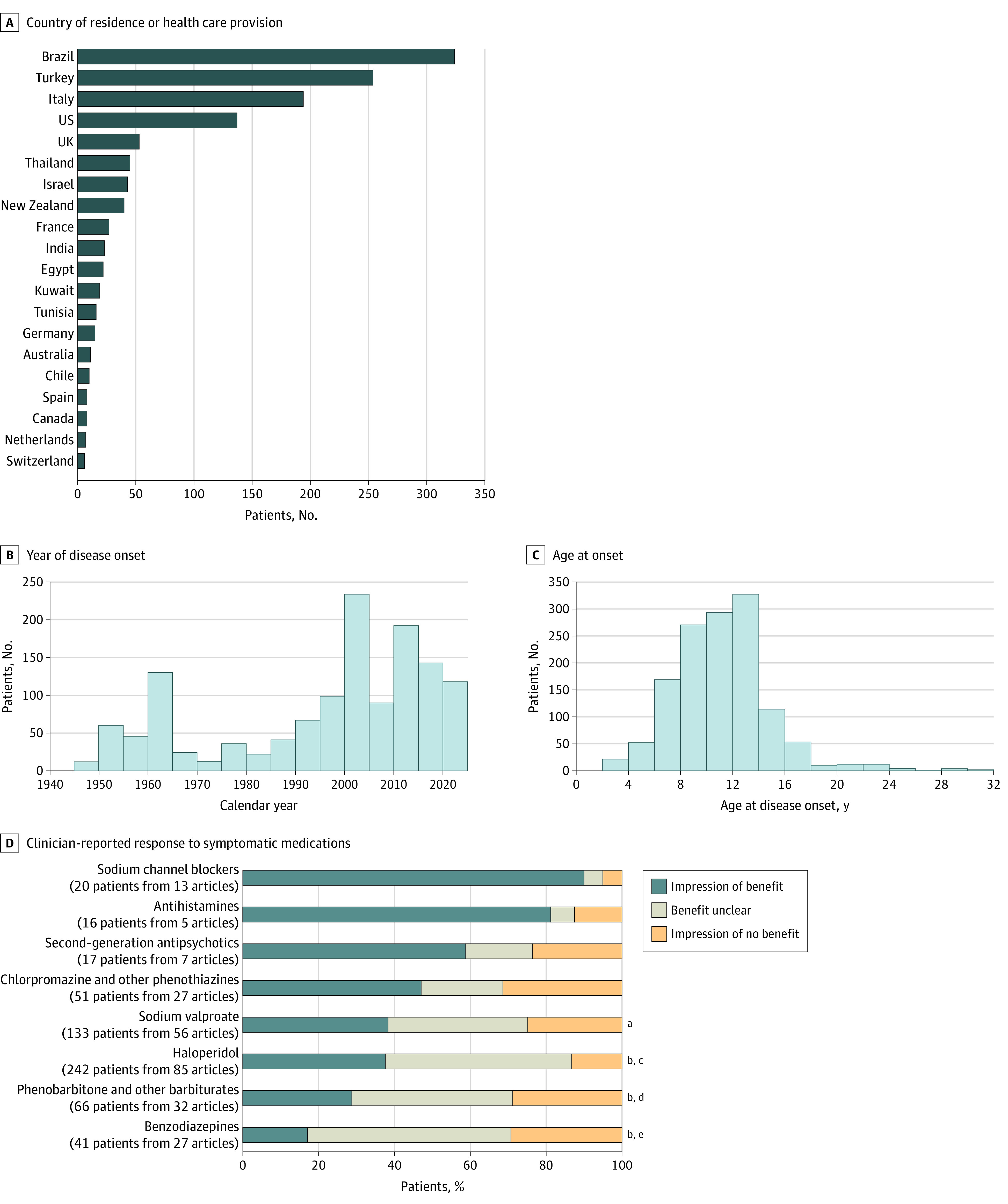
Patient Demographics and Clinician-Reported Response to Symptomatic Medications at the First Episode of Sydenham Chorea Data are shown for the first episode of Sydenham chorea in 1325 patients with disease onset since 1945. The top 20 countries of 50 total are shown. Data on year of onset were available in 416 patients and inferred from year of publication in the remaining. Seven patients with disease onset after 32 years of age are not shown. Significance indicated for comparisons of proportion with clinician-reported benefit in pairwise χ^2^ tests (Bonferroni-corrected). ^a^*P* = .001 vs sodium channel blockers. ^b^*P* < .001 vs sodium channel blockers. ^c^*P* = .04 vs antihistamines. ^d^*P* = .01 vs antihistamines. ^e^*P* < .001 vs antihistamines.

In total, 1325 patients from 50 countries had disease onset since 1945 (Table^332^; eTables 4-7 in Supplement 1; Figure 2A and B). Median (IQR) age was 10 (8-13) years in 1202 patients, and 1265 of 1317 patients (96.1%) were younger than 18 years (Figure 2C). Of 1272 patients, 875 (68.8%) were female and 397 (31.2%) were male. Of 1305 patients, 1111 (85.1%) were from populations considered at low risk for ARF; 33 of 435 (7.6%) had a family history of ARF. Of 442 patients, 27 (6.1%) had a history of other autoimmune or inflammatory diseases and 37 of 418 (8.9%) had preexisting psychiatric, neurologic, or neurodevelopmental disorders.

**Table.** Clinical Characteristics, Treatments, and Outcomes in 1325 Patients with SC Since 1945

Characteristic, Treatment, or Outcome	Patients, No./total No. (%)^a^
**Demographics and background history**	
Age at onset, y (n = 1202)	
Mean (SD)	10.9 (5.0)
Median (IQR)	10.3 (8.0-13.0)
Sex	
Female	875/1272 (68.8)
Male	397/1272 (31.2)
Family history of ARF	33/435 (7.6)
Preexisting psychiatric, neurologic, or neurodevelopmental disorders	37/418 (8.9)
Current economic classification of country of residence or health care provision^b^	
Income	
High	598/1317 (45.4)
Upper-middle	638/1317 (48.4)
Lower-middle	79/1317 (6.0)
Low	2/1317 (0.2)
Low population ARF risk	1111/1305 (85.1)
**Clinical features of the first SC episode (within first 3 mo after initial presentation)**	
Symptoms of infection preceding SC onset	259/485 (53.4)
Time between infection and SC onset, wk (n = 158/259)	
Mean (SD)	9.9 (12.1)
Median (IQR)	8.0 (3.0-14.0)
Antibiotics given before onset of ARF/SC	43/347 (12.4)
Hemichorea	208/664 (31.3)
Limb	572/574 (99.7)
Face	234/309 (75.7)
Trunk involvement	136/287 (47.4)
Impaired mobility	
Any	227/324 (70.1)
Severe: bedridden	38/324 (11.7)
Impaired speech	
Any	210/337 (62.3)
Severe: unable to speak	11/337 (3.3)
Impaired object manipulation	
Any	177/264 (67.0)
Severe: fully dependent for self-care and feeding	62/264 (23.5)
Impaired chewing or swallowing	
Any	57/251 (22.7)
Severe: nasogastric tube or parenteral feeding	16/251 (6.4)
Hypotonia	151/246 (61.4)
Motor impersistence (milkmaid’s grip)	66/125 (52.8)
Abnormal tongue movements (darting tongue)	53/146 (36.3)
Muscle weakness	63/196 (32.1)
Any psychiatric or behavioral symptom	312/484 (64.5)
Emotional lability	139/415 (33.5)
Fever	66/458 (14.4)
Worst mRS score (n = 460)	
Mean (SD)	3.2 (0.9)
Median (IQR)	3.0 (3.0-4.0)
Carditis or valvulitis	610/1151 (53.0)
Arthritis or arthralgia	275/1118 (24.6)
Erythema marginatum or subcutaneous nodules	31/836 (3.7)
**Investigation findings at the first SC episode (within first 3 mo after initial presentation)**	
ASOT elevated	393/547 (71.8)
Anti-DNase B elevated	80/134 (59.7)
Throat culture positive for GAS	68/182 (37.4)
Elevated ESR	255/470 (54.3)
Elevated CRP	94/340 (27.6)
ECG findings	
Prolonged PR interval	41/337 (12.2)
Any other abnormality	16/177 (9.0)
Abnormal brain structural MRI	
Any	43/225 (19.1)
Basal ganglia abnormal (focal swelling or T2/FLAIR hyperintensity)	16/223 (7.2)
White matter abnormal (focal T2/FLAIR hyperintensity)	15/224 (6.7)
Abnormal EEG findings (slow/disorganized activity and/or epileptic activity)	
Any	84/153 (54.9)
Focal or diffuse slow or disorganized activity	77/149 (51.7)
Epileptic activity (epileptic discharges or electrographic seizures)	7/147 (4.8)
Abnormal CSF findings	
Any	8/32 (25.0)
Pleocytosis ≥5 cells/uL (nonbloody tap only)	4/31 (12.9)
Intrathecal oligoclonal bands (present in CSF unmatched in serum)	2/26 (7.7)
**Treatment of the first SC episode**	
Antibiotics after onset of ARF/SC	
Any	744/867 (86.1)
IM penicillin G benzathine	591/782 (75.6)
Oral penicillin	68/770 (8.8)
Oral amoxicillin	12/770 (1.6)
Any immunotherapy given at first SC episode	231/898 (25.7)
Steroids	208/898 (23.2)
Any	56/882 (6.3)
IV methylprednisolone	40/867 (4.6)
IM ACTH	16/867 (1.8)
Oral steroids	
Any	167/881 (19.0)
Prednisone	111/848 (13.1)
Deflazacort or dexamethasone	4/845 (0.5)
Duration of IV and oral steroid treatment at first episode, wk (n = 136/208)	
Mean (SD)	6.9 (8.8)
Median (IQR)	4.0 (2.4-8.0)
IVIG	21/898 (2.3)
Plasma exchange	12/898 (1.3)
Time between SC symptom onset and first IT, d (n = 135/231)	
Mean (SD)	38.1 (86.5)
Median (IQR)	17.0 (10.0-30.0
Symptomatic pharmacological treatments given at first episode	
Any	540/687 (78.6)
Haloperidol	241/663 (36.3)
Valproate	136/663 (20.5)
Phenobarbital	62/663 (9.4)
Total weeks of symptomatic treatments at first episode (n = 169/540)	
Mean (SD)	18.9 (80.6)
Median (IQR)	8.0 (4.0-14.0)
**Clinical course and final follow-up**	
Full resolution of chorea after the first SC episode	529/622 (85.0)
Time from initial SC onset to initial full resolution of chorea, mo (n = 353/529)	
Median (IQR)	3.0 (1.2-6.0)
Time from initial SC onset to final follow-up, mo (n = 720)	
Mean (SD)	36.6 (100.7)
Median (IQR)	12.0 (5.0-24.3)
Relapse of SC	263/766 (34.3)
Ongoing chorea at final follow-up	138/595 (23.2)
Any psychiatric or behavioral symptoms at final follow-up	28/472 (5.9)
mRS score at final follow-up (n = 203)^c^	
Mean (SD)	0.3 (0.7)
Median (IQR)	0
0	153 (75.4)
1	34 (16.7)
2	13 (6.4)
3	3 (1.5)

Abbreviations: ACTH, Adrenocorticotropic hormone; ARF, acute rheumatic fever; ASOT, antistreptolysin O titer; CRP, C-reactive protein; CSF, cerebrospinal fluid; ECG, electrocardiography; EEG, electroencephalography; ESR, erythrocyte sedimentation rate; FLAIR, Fluid attenuated inversion recovery; GAS, group A streptococcus; IM, intramuscular; IT, immunotherapy; IV, intravenous; IVIG, intravenous immunoglobulin; MRI, magnetic resonance imaging; mRS, modified Rankin Scale; SC, Sydenham chorea.

^a^ Descriptive data are provided for patients with available information, hence the varying denominators.

^b^ Data retrieved from The World Bank on January 22, 2024.^332^

^c^ Included only patients with mRS 0 to 1 at any time and patients with mRS 2 or higher with 6 months or longer follow-up from last SC event.

### Clinical Features of the First Episode of Sydenham Chorea

Preceding symptoms of infection were reported in 259 of 485 patients (53.4%). Initial presenting SC symptoms were motor in 325 of 405 patients (80.2%), psychiatric or behavioral in 27 of 405 patients (6.7%) and both combined in 53 of 405 patients (13.1%). Chorea involved the limbs in 572 of 574 patients (99.7%), face in 234 of 309 patients (75.7%), and trunk in 136 of 287 patients (47.4%); 208 of 664 patients (31.3%) had hemichorea. Of 334 patients, 227 (70.1%) had impaired mobility, 177 of 264 patients (67.0%) had impaired object manipulation, 210 of 337 patients (62.3%) had impaired speech, and 57 of 251 patients (22.7%) had impaired chewing or swallowing. Of 484 patients, 312 (64.5%) had psychiatric or behavioral symptoms, most frequently emotional lability, anxiety, irritability, hyperactivity, and aggressive behavior (eFigure 2 in Supplement 1). Mental health specialist assessment was reported in 57 of 324 patients (17.6%), specific assessment tools in 42 of 321 patients (13.1%), and assignment of a formal psychiatric diagnosis in 31 of 312 patients (9.9%). In total, 452 of 498 patients (90.8%) were hospitalized. The median (IQR) mRS score at nadir was 3 (3-4) in 460 patients; 139 of 460 patients (30.2%) had severe disease (mRS 4-5), including 28 of 234 patients (12.0%) with mRS score of 5 and complete loss of self-care skills. Other major manifestations of ARF included carditis or valvulitis in 610 of 1151 patients (53.0%), arthritis or arthralgia in 275 of 1118 patients (24.6%), and skin manifestations in 31 of 836 patients (3.7%).

### Findings at the First Episode of Sydenham Chorea

Evidence of preceding streptococcal infection was reported in 559 of 655 patients (85.3%)^2^: elevated antistreptolysin O titer in 393 of 547 patients (71.8%), elevated anti-DNase B titer in 80 of 134 patients (59.7%), and GAS present in the throat culture for 68 of 182 patients (37.4%). Of 470 patients, 255 (54.3%) had elevated erythrocyte sedimentation rate, and 94 of 340 patients (27.6%) had elevated C-reactive protein (Table). Of 337 patients, 41 (12.2%) had a prolonged PR interval, and 16 of 177 patients (9.0%) had other electrocardiographic abnormalities. Of 225 patients, 43 (19.1%) had findings on brain magnetic resonance imaging, including 16 of 223 (7.2%) showing abnormal basal ganglia and 15 of 224 (6.7%) showing abnormal white matter. Of 153 patients, 84 (54.8%) had abnormal findings on electroencephalograms: 77 of 149 (51.7%) with slow or disorganized background activity and 7 of 147 (4.8%) with discharges or seizures. Of 32 patients, 8 (25.0%) had abnormal findings in cerebral spinal fluid, including 4 of 31 (12.9%) with pleocytosis and 2 of 26 (7.7%) with intrathecal oligoclonal bands; 0 of 30 patients had elevated CSF protein.

### Treatment of the First Episode of Sydenham Chorea

Antibiotics were used as treatment in 744 of 867 patients (86.1%), and immunotherapy in 231 of 898 patients (25.7%): 208 of 898 patients (23.2%) received corticosteroids, 21 of 898 patients (2.3%) received intravenous immunoglobulin, and 12 of 898 patients (1.3%) received plasma exchange. Of 165 patients, 9 (5.5%) had adverse events associated with immunotherapy (eTable 5 in Supplement 1). Of 687 patients, 540 (78.6%) received symptomatic pharmacological treatments, including haloperidol for 241 of 663 patients (36.3%) and sodium valproate for 136 of 663 patients (20.5%). Clinician-reported benefit was most frequent for sodium channel blockers (carbamazepine in 18 patients, phenytoin in 2 patients; 18 of 20 patients [90.0%] with benefit) and antihistamines (hydroxyzine in 13 patients, diphenhydramine in 3 patients; 13 of 16 patients [81.2%] with benefit) (Figure 2D and eTable 6 in Supplement 1). Of 408 patients, 36 (8.8%) had adverse events associated with symptomatic treatments, including 23 (5.6%) with severe adverse events (attributed to haloperidol in 13 patients, chlorpromazine and other phenothiazines in 9 patients, and sodium valproate in 1 patient) (eTable 7 in Supplement 1).

### Descriptive Data on Disease Course and Functional Outcome

Median (IQR) duration of follow-up was 12 (5-37) months for 720 patients. There were no deaths.

#### Chorea Duration and Disease Course

**Figure 3.**
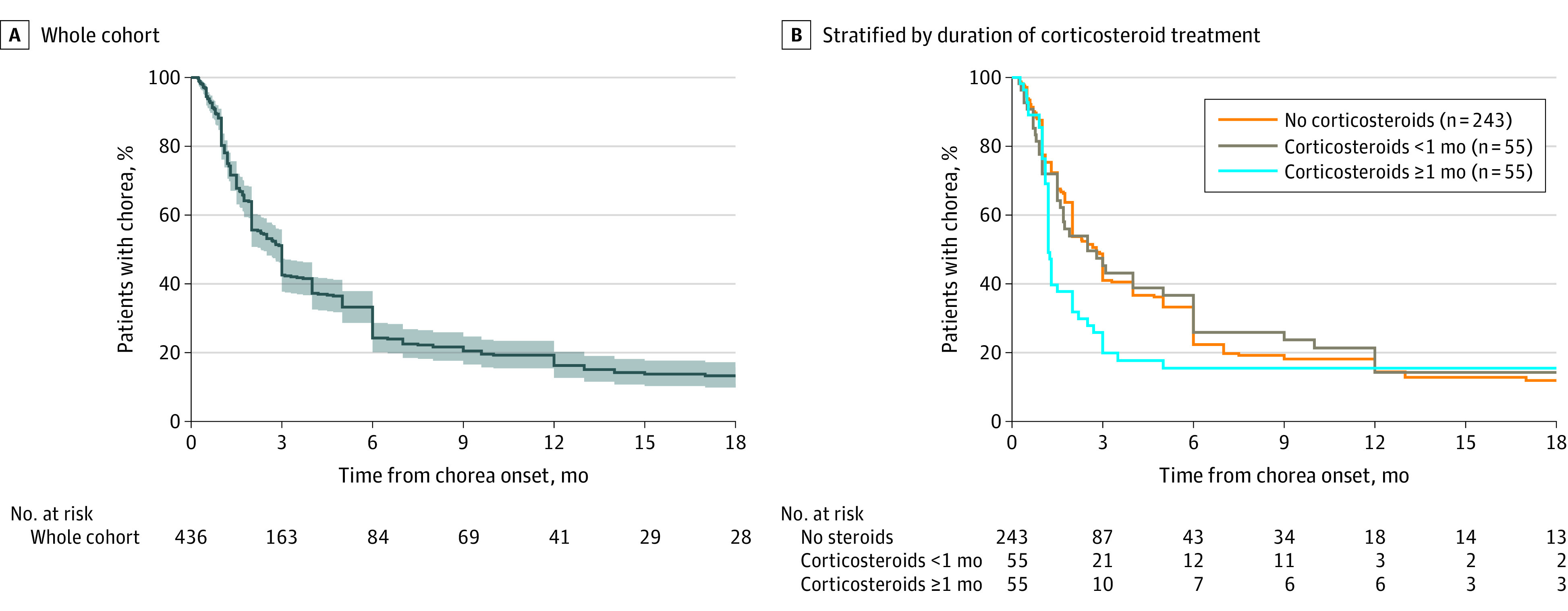
Time to Chorea Resolution at the First Episode of Sydenham Chorea Shading indicates 95% CIs.

Of 622 patients, 529 (85.0%) had full resolution of chorea at the first episode. The median (IQR) chorea duration was 3.0 (1.2-6.0) months (Figure 3A). Including all patients with available data on disease course, 263 of 766 patients (34.3%) experienced relapse; among them 171 of 240 (71.3%) relapsed once, 47 of 240 (19.6%) relapsed twice, 15 of 240 (6.3%) relapsed 3 times, and 7 of 240 (2.9%) experienced relapse 4 or more times (maximum 8 times). Median (IQR) interval to first relapse was 16.0 (8.3-48.0) months in 189 patients. Evidence of GAS infection was reported in 31 of 98 patients (31.6%) compared with 559 of 655 (85.3%) at the initial episode (*P* < .001).

#### Functional Outcome

Including all patients with available follow-up data, 138 of 595 patients (23.2%) had ongoing chorea at final follow-up, 28 of 472 patients (5.9%) had ongoing psychiatric or behavioral symptoms, and 12 of 395 patients (3.0%) had ongoing cognitive or school performance problems. Among patients with a final follow-up of 6 or more months after the last SC episode (or final mRS score of 0-1 at any time), 187 of 203 (92.1%) had an mRS score of 0 or 1 at final follow-up (median, 0; range, 0-3).

### Clinical and Treatment Factors Associated With Disease Course and Outcome

#### Chorea Duration

**Figure 4.**
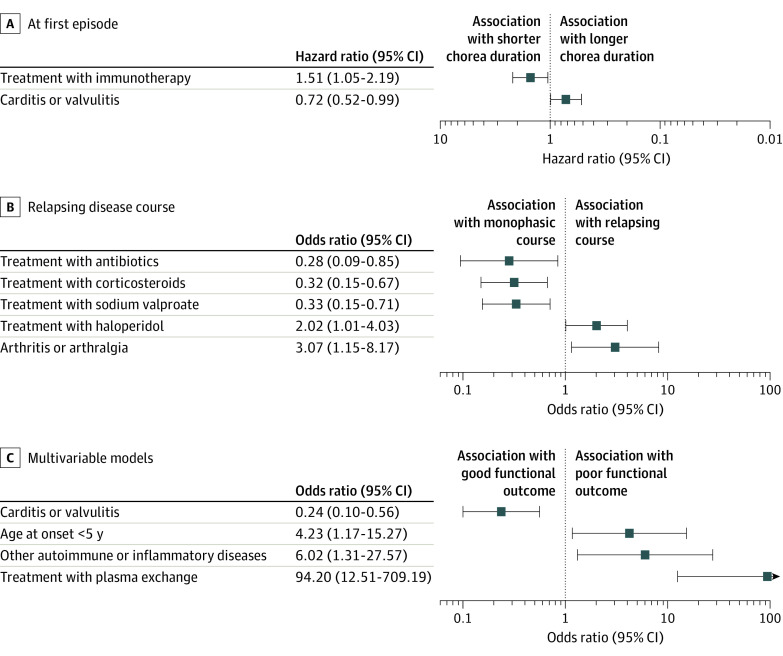
Independent Associations of Clinical and Treatment Factors With Disease Course and Outcome Data are shown for variables significant at *P* < .05 in the Cox proportional hazards regression model (A) and logistic regression models (B, C).

We included 178 patients in the model for chorea duration at first episode (Figure 4A; eTable 8 in Supplement 1). Immunotherapy was associated with shorter chorea duration (hazard ratio [HR] for chorea resolution during treatment 1.51 [95% CI, 1.05-2.19]; *P* = .03). Carditis or valvulitis was associated with longer chorea duration (HR, 0.72 [95% CI, 0.52-0.99]; *P* = .04). The median chorea duration in 55 patients treated with 1 or more months of steroids was 1.2 months (95% CI, 1.2-2.0) vs 2.8 months (95% CI, 2.0-3.0) for 243 patients not treated with steroids (Tarone-Ware test *P* = .004; log-rank test *P* = .02). The median chorea duration for 55 patients treated for less than 1 month with steroids was 2.5 months (95% CI, 1.5-5.0), not significantly different from the groups with 1 or more months of treatment (*P* = .08) or no steroid treatment (*P* = .84) (Figure 3B).

#### Disease Course

We included 345 patients in the model for relapsing disease course (263 with relapse) (Figure 4B; eTable 8 in Supplement 1). Factors associated with relapsing course were arthritis or arthralgia (odds ratio [OR], 3.07 [95% CI, 1.15-8.17]; *P* = .02) and treatment with haloperidol at the first episode (OR, 2.02 [95% CI, 1.01-4.03]; *P* = .046). Factors associated with monophasic course were treatment with antibiotics (OR, 0.28 [95% CI, 0.09-0.85]; *P* = .02), corticosteroids (OR, 0.32 [95% CI, 0.15-0.67]; *P* = .003), or sodium valproate (OR, 0.33 [95% CI, 0.15-0.71]; *P* = .004). Compared with the odds for 184 patients not treated with steroids, the odds of experiencing a relapsing course were significantly lower (OR, 0.10 [95% CI, 0.04-0.25]; *P* < .001) among 33 patients treated for 1 or more months with steroids and significantly lower (OR, 0.31 [95% CI, 0.10-0.97]; *P* = .03) among 17 patients treated for less than 1 month with steroids. There was no significant difference in relapsing disease course between the treatment groups of less than 1 month and 1 or more months (OR, 0.34 [95% CI, 0.08-1.34]; *P* = .12).

#### Functional Outcome

We included 338 patients in the model for functional outcome (47 patients [13.9%] with poor outcome) (Figure 4C; eTable 8 in Supplement 1). Factors associated with poor outcome were treatment with plasma exchange (OR, 94.2 [95% CI, 12.51-709]; *P* < .001), history of other autoimmune or inflammatory diseases (OR, 6.02 [95% CI, 1.31-27.57]; *P* = .02) and younger age (<5 years) at disease onset (OR, 4.23 [95% CI, 1.17-15.27]; *P* = .03). Carditis or valvulitis was associated with good outcome (OR, 0.24 [95% CI, 0.10-0.56]; *P* = .001).

## Discussion

To our knowledge, this individual patient data meta-analysis is the most comprehensive evidence synthesis to date for SC, including IPD from 1479 cases. We found that immunotherapy, in particular with corticosteroids, was associated with faster resolution of chorea at the first episode, and that antibiotics, corticosteroids, and sodium valproate were associated with lower rates of relapse, which occurred in 34.3% of patients overall. We found that 86.1% of patients had a good final functional outcome, but no treatment factors were identified in association with this.

Sydenham chorea was one of the earliest conditions recognized in neurology, and its distinctive features, including the full rheumatic syndrome recognized since 1889,^333^ give us some confidence that the disorder has been relatively consistently identified over time. We found that age at onset (median 10 years) and female preponderance (2.2:1)^12^ have remained almost constant for more than 100 years, while the frequencies of fever, arthritis or arthralgia, and poor functional outcome have reduced in the modern era (Figure 1). In the modern era, 12.0% of patients had complete loss of upper and lower limb function (possibly consistent with chorea paralytica). Psychiatric or behavioral symptoms were reported in 64.5% of patients, with formal psychiatric diagnoses (including attention-deficit/hyperactivity disorder or obsessive-compulsive disorder) assigned in 9.9% of patients. However, mental health specialist assessment was reported in only 17.6% of cases. In cohorts undergoing standardized psychiatric evaluations, rates of attention-deficit/hyperactivity disorder up to 31% and obsessive-compulsive disorder up to 24% have been reported.^8,334^

The median duration of chorea at the first episode was 3 months (Figure 3A), longer than reported in the era before 1945 and rising to 4 months in recent decades (Figure 1), perhaps due to increased recognition of subtle or fluctuating chorea. Immunotherapy was associated with shorter chorea duration, with significantly higher HR for chorea resolution during treatment (Figure 4A). Further analysis stratified by steroid treatment duration showed significantly shorter chorea duration (median 1.2 months) for patients receiving steroids for 1 or more months (Figure 3B). Our finding of benefit for steroids in hastening resolution of the acute SC episode is consistent with previous observational studies,^5,7,11,17,335,336,337,338,339,340,341^ and the only placebo-controlled RCT to date, in which 22 children receiving prednisone had a mean (SD) chorea duration of 1.8 (0.8) months vs 3.9 (2.8) months for placebo.^15^ Although we were unable to evaluate intravenous immunoglobulin specifically due to small numbers, this treatment has also been shown in an RCT to reduce the duration of symptomatic treatment required.^16^ The only factor associated with longer chorea duration in the present study was carditis or valvulitis, with a reduced HR for chorea resolution of 0.72. Carditis may indicate greater inflammatory activity and more severe disease^6^; a recent report similarly identified arthritis as a risk factor for longer chorea duration,^17^ although this finding was not replicated in the present study.

Symptomatic medications were used in 78.6% of patients. We did not find any associations with chorea duration; however, clinicians often reported benefit, most frequently for treatment with sodium channel blockers, such as carbamazepine,^342,343^ antihistamines, and second-generation antipsychotics (Figure 2D; eTable 6 in Supplement 1), none of which were associated with severe adverse events in the present study (eTable 7 in Supplement 1). Haloperidol was less frequently associated with benefit and more frequently associated with severe adverse events such as hypertonia or parkinsonism. In 1 study, 23% of SC patients receiving haloperidol required treatment change due to adverse effects^344^; SC has been hypothesized to be a risk factor for drug-induced parkinsonism.^345^

Relapse was reported in 34.3% of patients overall, similar to previous studies.^9,10,11^ We found that antibiotic treatment was associated with significantly reduced odds of relapsing course (Figure 4B),^346^ and as we were unable to account for treatment adherence, the actual benefit may exceed this value.^7,9,12^ Treatment with corticosteroids at the first episode was associated with 3.1-fold reduced odds of relapsing course. Although this association has been suggested in previous studies, it has not been previously observed with statistical significance.^5,335,340^ Additional analysis confirmed that even steroid courses for less than 1 month were associated with monophasic disease course. Unexpectedly, treatment with sodium valproate was also associated with reduced odds of relapsing course. Valproate is regarded as a safe and efficacious treatment for symptom relief in SC^342,344,347^ but has not been previously associated with protection against relapse. Valproate is a histone deacetylase inhibitor that can induce epigenetic modifications to immune cells; in an ex vivo study of monocyte-derived macrophages from patients with systemic lupus erythematosus, valproate upregulated anti-inflammatory macrophages and cytokines while downregulating proinflammatory macrophages and tumor necrosis factor α.^348^ In animal models, valproate reduces inflammation in the optic nerve and spinal cord.^349,350^ As epigenetic modifications can be long-lasting, it is plausible that valproate could reduce relapse risk in SC; however, the proposed anti-inflammatory mechanisms are speculative and require further study. Conversely, treatment with haloperidol was associated with increased odds of relapsing course. It has been suggested that some SC recurrences may reflect a persisting susceptibility to movement disorder rather than true relapses of ARF,^9,351^ and indeed, in the present study, evidence of GAS infection was less frequent at recurrence (31.6%) than at presentation (85.3%). One possibility is that some patients treated with haloperidol had such a susceptibility due to baseline differences (eg, worse disease severity or lower-resource health care settings), which we were unable to control for in our multivariable model. Another possibility is that haloperidol may induce long-term basal ganglia changes (as observed in first-episode psychosis^352^), which could confer future susceptibility to dyskinesia in some patients, although this hypothesis remains to be adequately explored.

Poor functional outcome occurred in 13.9% of patients. There is a well-described group of patients who develop chronic disease with persistent chorea or psychiatric symptoms^1,6,7,8^ despite no evidence of immunological difference from patients in remission and no structural brain injury, although abnormalities suggestive of neuronal loss in the basal ganglia have been reported from magnetic resonance spectroscopy.^353,354^ We found that patients with poor outcome were more likely to be younger than 5 years at onset, undergo plasma exchange, and have comorbid autoimmune or inflammatory disorders; they were also less likely to have carditis or valvulitis (Figure 4C). Plasma exchange is rarely used in SC and is mainly used as rescue therapy after failure of other treatments.^355^ Hence, the association with poor outcome likely reflects a severity bias, which our main severity measure (mRS score ≥4) may not capture. Other treatments were not significantly associated with long-term outcome in the present study; however a previous RCT showed benefit for intravenous immunoglobulin therapy in more subtle functional outcome measures.^356^ The association of carditis with good functional outcome is contrary to previous studies of prolonged SC^6^ and remains to be explained; it could be that some patients in the poor outcome group had additional or alternative neurological disorders that were not associated with carditis.

### Limitations

The main limitations of this meta-analysis were the retrospective nature of the data, inclusion of articles only in a subset of languages, underrepresentation of low and lower-middle income countries (Table), and inclusion of case reports that were susceptible to diagnostic error (especially in older cohorts, when, for example, N-methyl-d-aspartate receptor antibody testing was not available) and reporting biases, such as reporting patients with worse disease, atypical features (eg, abnormal findings on magnetic resonance imaging), or atypical treatment response. The data on clinician-reported benefit from symptomatic medications may be especially subject to such biases, and the medications evaluated as most beneficial in this analysis were given to relatively small numbers of patients. Estimates of feature frequencies may be biased by underreporting of negative findings or conversely by underreporting of more subtle positive findings (eg, individual psychiatric symptoms). Adverse treatment effects were also likely underreported. Data collected were limited by heterogeneous availability, hence hot-deck imputation was used to enable multivariable analysis. Although this method generates clinically plausible values (by constraining imputation to values already present in the database), it does not guarantee complete extinction of bias, as implicit assumptions are required in the choice of metric to match donors to recipients.^23^ As data were missing not at random, sensitivity analyses were conducted for year of onset and missingness (eMethods 3, eTables 9-17 in Supplement 1); however, the main findings for immunotherapy, corticosteroids, and valproate were supported by sensitivity analyses on reduced data sets (eTables 9-17 in Supplement 1). Functional outcome evaluation as good vs poor was a pragmatic grouping of different outcome measures (mRS, persisting chorea, or psychiatric or behavioral symptoms), necessary to enable analysis of heterogeneously reported data. We acknowledge that this grouping may oversimplify important patient differences, and the predictor variables may not capture all patient complexities, potentially explaining some associations between treatments and adverse outcomes.

## Conclusions

This meta-analysis found evidence to support the use of immunotherapy, in particular corticosteroids, to reduce the duration of chorea at the first episode of SC and to support the use of antibiotics, corticosteroids, and sodium valproate to reduce the risk of relapse, although the mechanism of action for valproate is not fully understood and requires further investigation. Most patients achieved a good final functional outcome; however, specific treatment factors associated with this outcome remain unknown. This synthesis should help direct future research questions and is forming the base for an ongoing international effort with Delphi methodology to provide consensus-based recommendations for the management of SC.

## Notes

Supplement 1. **eMethods 1.** Literature Search and Data Collection **eMethods 2.** Statistical Analysis **eMethods 3.** Sensitivity Analysis **eFigure 1.** PRISMA Flow Diagram **eFigure 2.** Symptom Frequencies Among Patients With Reported Psychiatric/Behavioural Symptoms **eTable 1.** Literature Search and Record Selection: Databases Searched and Numbers of Records Retrieved, Screened and Included **eTable 2.** Proportions of Missing (Imputed) Data in the Multivariable Models **eTable 3.** Historical Comparison of Patients With Disease Onset Before and After 1945 **eTable 4.** Complete Descriptive Data in 1325 Patients with Sydenham’s Chorea Since 1945 **eTable 5.** Adverse Events Associated With Immunotherapy **eTable 6.** Clinician-Reported Benefit From Symptomatic Medications **eTable 7.** Adverse Events Associated With Symptomatic Medications **eTable 8.** Multivariable Model Results **eTable 9.** Data Missingness According to Year of Disease Onset **eTable 10.** Nested Model for Chorea Duration at First Episode With Twentieth Century Cases Withheld **eTable 11.** Nested Model for Relapsing Disease Course With Twentieth Century Cases Withheld **eTable 12.** Nested Model for Poor Functional Outcome With Twentieth Century Cases Withheld **eTable 13.** Outcome Distributions for Variables With High Missingness in the Chorea Duration at First Episode Model **eTable 14.** Outcome Distributions for Variables With High Missingness in the Relapsing Disease Course Model **eTable 15.** Outcome Distributions for Variables With High Missingness in the Poor Functional Outcome Model **eTable 16.** Nested Model for Chorea Duration at First Episode With Cases Missing Data in ≥1 Missing Not at Random (MNAR) Variables Withheld **eTable 17.** Nested Model for Poor Functional Outcome with Cases Missing Data in ≥1 Missing Not at Random (MNAR) Variables Withheld

Supplement 2. **Nonauthor Collaborators.** Members of the Sydenham’s Chorea Systematic Literature Review Working Group

Supplement 3.
Data Sharing Statement
