# Supplementary material for: Treatments and Outcomes Among Patients with Sydenham Chorea: A Meta-Analysis
Source: JAMA Netw Open. 2024 Apr 16;7(4):e246792. doi: 10.1001/jamanetworkopen.2024.6792 (PMC11022117; doi:10.1001/jamanetworkopen.2024.6792)
Supplement: Supplement 1. — eMethods 1. Literature Search and Data Collection eMethods 2. Statistical Analysis eMethods 3. Sensitivity Analysis eFigure 1. PRISMA Flow Diagram eFigure 2. Symptom Frequencies Among Patients With Reported Psychiatric/Behavioural Symptoms eTable 1. Literature Search and Record Selection: Databases Searched and Numbers of Records Retrieved, Screened and Included eTable 2. Proportions of Missing (Imputed) Data in the Multivariable Models eTable 3. Historical Comparison of Patients With Disease Onset Before and After 1945 eTable 4. Complete Descriptive Data in 1325 Patients with Sydenham’s Chorea Since 1945 eTable 5. Adverse Events Associated With Immunotherapy eTable 6. Clinician-Reported Benefit From Symptomatic Medications eTable 7. Adverse Events Associated With Symptomatic Medications eTable 8. Multivariable Model Results eTable 9. Data Missingness According to Year of Disease Onset eTable 10. Nested Model for Chorea Duration at First Episode With Twentieth Century Cases Withheld eTable 11. Nested Model for Relapsing Disease Course With Twentieth Century Cases Withheld eTable 12. Nested Model for Poor Functional Outcome With Twentieth Century Cases Withheld eTable 13. Outcome Distributions for Variables With High Missingness in the Chorea Duration at First Episode Model eTable 14. Outcome Distributions for Variables With High Missingness in the Relapsing Disease Course Model eTable 15. Outcome Distributions for Variables With High Missingness in the Poor Functional Outcome Model eTable 16. Nested Model for Chorea Duration at First Episode With Cases Missing Data in ≥1 Missing Not at Random (MNAR) Variables Withheld eTable 17. Nested Model for Poor Functional Outcome with Cases Missing Data in ≥1 Missing Not at Random (MNAR) Variables Withheld [file jamanetwopen-e246792-s001.pdf]

Supplementary Online Content

Eyre M, Thomas T, Ferrarin E, et al. Treatments and outcomes among patients with Sydenham chorea: a meta-analysis. *JAMA Netw Open*. 2024;7(4):e246792. doi:10.1001/jamanetworkopen.2024.6792

eMethods 1. Literature Search and Data Collection
eMethods 2. Statistical Analysis
eMethods 3. Sensitivity Analysis
eFigure 1. PRISMA Flow Diagram
eFigure 2. Symptom Frequencies Among Patients With Reported Psychiatric/Behavioural Symptoms
eTable 1. Literature Search and Record Selection: Databases Searched and Numbers of Records Retrieved, Screened and Included
eTable 2. Proportions of Missing (Imputed) Data in the Multivariable Models
eTable 3. Historical Comparison of Patients With Disease Onset Before and After 1945
eTable 4. Complete Descriptive Data in 1325 Patients with Sydenham's Chorea Since 1945
eTable 5. Adverse Events Associated With Immunotherapy
eTable 6. Clinician-Reported Benefit From Symptomatic Medications
eTable 7. Adverse Events Associated With Symptomatic Medications
eTable 8. Multivariable Model Results
eTable 9. Data Missingness According to Year of Disease Onset
eTable 10. Nested Model for Chorea Duration at First Episode With Twentieth Century Cases Withheld
eTable 11. Nested Model for Relapsing Disease Course With Twentieth Century Cases Withheld
eTable 12. Nested Model for Poor Functional Outcome With Twentieth Century Cases Withheld
eTable 13. Outcome Distributions for Variables With High Missingness in the Chorea Duration at First Episode Model
eTable 14. Outcome Distributions for Variables With High Missingness in the Relapsing Disease Course Model
eTable 15. Outcome Distributions for Variables With High Missingness in the Poor Functional Outcome Model
eTable 16. Nested Model for Chorea Duration at First Episode With Cases Missing Data in ≥1 Missing Not at Random (MNAR) Variables Withheld
eTable 17. Nested Model for Poor Functional Outcome with Cases Missing Data in ≥1 Missing Not at Random (MNAR)Variables Withheld

This supplementary material has been provided by the authors to give readers additional information about their work.

eMethods 1. Literature Search and Data Collection

Individual patient data (IPD) was extracted from the retrieved articles by four paediatric neurologists (ME, TT, SK, MN) and a research support pharmacist (EF). Minimum IPD availability for inclusion was diagnosis as Sydenham's chorea (SC) by the authors in association with at least one of the following: availability of data on gender AND/OR age at onset AND/OR clinical symptoms at onset AND/OR treatment data. Cases were excluded if the data extractor assessed the diagnosis of SC to be likely incorrect. Data were collected using a standardised proforma including patient demographics; population acute rheumatic fever (ARF) risk (defined as moderate-to-high for patients from countries with rheumatic heart disease prevalence >1 per 1000 or from high-risk communities in any country);[1,2] pre-existing inflammatory/neuropsychiatric disorders; infectious symptoms preceding SC onset; neurological and psychiatric/behavioural signs and symptoms at first SC episode; psychiatric/behavioural evaluation including use of specific assessments tools and assignment of formal psychiatric diagnoses (e.g. DSM/ICD-10 disorder); disease severity at first episode (scored with the modified Rankin Scale, mRS);[3] other major manifestations of ARF (carditis/valvulitis, joint involvement, skin manifestations);[1] laboratory investigations at first episode including evidence of preceding group A streptococcal (GAS) infection[1] and CSF abnormalities; neuroimaging and EEG findings at first episode; antibiotic treatment; immunotherapies; symptomatic medications and the treating clinician's impression of their effect; adverse events associated with immunotherapies and symptomatic medications (graded according to the National Institutes of Health Common Terminology Criteria for Adverse Events [CTCAE] version 5.0); time to chorea resolution at first episode; any relapses (as defined by the article authors) and their timing; and final outcome including persisting symptoms and mRS score at final follow-up. Abnormal CSF was defined as pleocytosis ≥5 cells/uL in a non-bloody tap, intrathecal unmatched oligoclonal bands, increased CSF IgG index or protein ≥45 mg/dL. Abnormal MRI was defined as brain parenchymal swelling or increased T2 or fluid-attenuated inversion recovery (FLAIR) signal intensity. Abnormal EEG was defined as focal or diffuse slow/disorganised background activity, electrographic seizures or interictal epileptiform discharges. When not reported in the original article, mRS was retrospectively assigned following review of adequate clinical data provided.

eMethods 2: Statistical Analysis

Hot-deck imputation was performed prior to multivariable modelling.[4,5] Hot deck methods impute missing values within a database by using available values (donors) from the same database. No limit was set on the number of single donor selections. Case distance was weighted by univariate association of each predictor variable with the target outcome variable (time to chorea resolution at the first episode, relapsing disease course or functional outcome). Missing values were filled sequentially, starting with the variable with minimum frequency of missingness (reference imputation) (**eTable 2**). Outcome variables were not imputed. Following imputation, nonbinary predictor variables were recoded: worst mRS score was binarised at mRS ≥4, and age was categorised as preschool (<5 years), school age (5-11 years), adolescent (12-17 years) or adult (≥18 years), with 5-11 years as the reference category. In the Cox proportional hazard model, symptomatic medications and immunotherapy were included as time-varying features. As time-varying data (i.e. treatment start and stop times) were not always available for each treatment individually, patients treated with multiple symptomatic medications were excluded from this model, and immunotherapies (corticosteroids, IVIG and/or plasma exchange) were combined into a single time-varying feature. A single course of IVIG was regarded as having a treatment duration of one month. Time-varying features were not imputed. If a regression model failed to converge due to matrix singularity, predictor variables with zero or near-zero variance in one or both outcome classes (due to insufficient numbers of patients with the feature present) were dropped until convergence was achieved. Adjusted odds or hazard ratios were derived from the model coefficients. In the univariate survival analysis stratified by corticosteroid duration, the Tarone-Ware test was used as the primary analysis to give more weight to earlier events of chorea resolution, i.e. those which occurred during the period when patients were either receiving or not receiving steroids; the findings from this primary analysis were then validated with the log-rank test.

eMethods 3. Sensitivity Analysis

To evaluate potential biases in the regression models, secular trends were described and sensitivity analyses conducted with respect to year of disease onset and data missingness. For year of onset the dataset (cases included in ≥1 of the three multivariable models, n=618) was split by the median year of onset (2000). Missingness was compared in cases with onset before and since the year 2000 using Fisher's exact test (**eTable 9**) and nested regression models were created for each of the three outcomes, in which cases with pre-2000 onset were withheld (**eTables 10-12**). In order to test for differences in outcomes between cases with missing and complete data, the outcome distributions were compared between cases with missing data (inner distribution) and complete data (outer distribution) for each variable with missingness ≥20% in each model; variables with significant differences in the distributions (p<0.05 in Fisher's exact test for binomial outcomes or Kaplan-Meier survival analysis [log-rank test] for the

© 2024 Eyre M et al. *JAMA Network Open.*

chorea duration outcome) are shown in **eTables 13-15**. Where significant differences were found (i.e. data were missing not at random [MNAR] with respect to the outcome) we created nested models in which cases with missing data in ≥1 of the MNAR variables for that outcome were withheld (**eTables 16-17**), except for relapsing disease course, in which only 37 cases remained after exclusion, insufficient to fit a nested model.

eReferences

1. Gewitz MH, Baltimore RS, Tani LY, et al. Revision of the Jones Criteria for the diagnosis of acute rheumatic fever in the era of Doppler echocardiography: a scientific statement from the American Heart Association. *Circulation*. 2015;131(20):1806-1818. doi:10.1161/CIR.0000000000000205
2. Watkins DA, Johnson CO, Colquhoun SM, et al. Global, Regional, and National Burden of Rheumatic Heart Disease, 1990-2015. *N Engl J Med*. 2017;377(8):713-722. doi:10.1056/NEJMoa1603693
3. van Swieten JC, Koudstaal PJ, Visser MC, Schouten HJ, van Gijn J. Interobserver agreement for the assessment of handicap in stroke patients. *Stroke*. 1988;19(5):604-607. doi:10.1161/01.str.19.5.604
4. Andridge RR, Little RJA. A Review of Hot Deck Imputation for Survey Non-response. *Int Stat Rev*. 2010;78(1):40-64. doi:10.1111/j.1751-5823.2010.00103.x
5. Nosadini M, Eyre M, Molteni E, et al. Use and Safety of Immunotherapeutic Management of N-Methyl-d-Aspartate Receptor Antibody Encephalitis: A Meta-analysis. *JAMA Neurol*. 2021;78(11):1333-1344. doi:10.1001/jamaneurol.2021.3188

© 2024 Eyre M et al. *JAMA Network Open.*

eFigure 1. PRISMA Flow Diagram

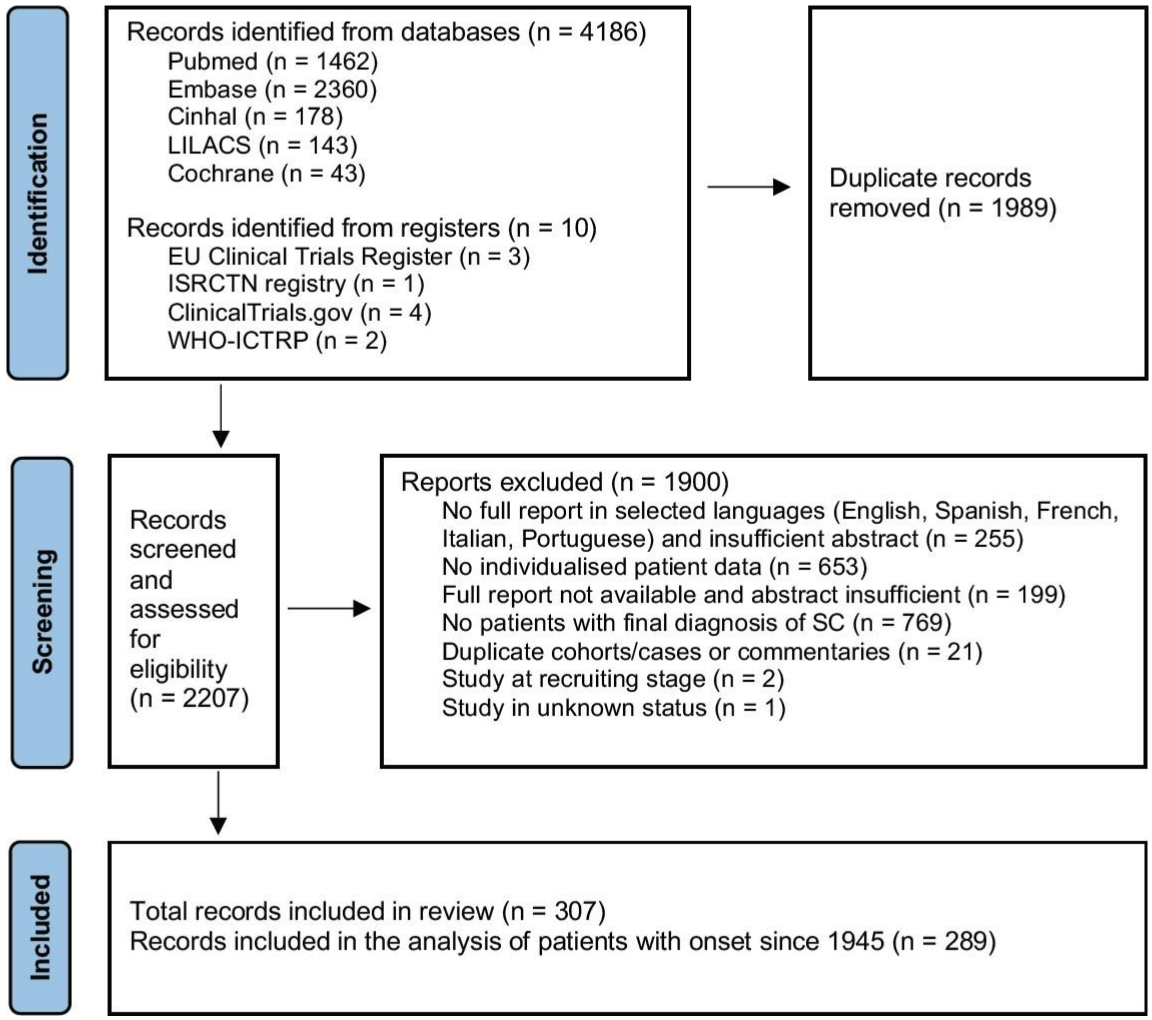

© 2024 Eyre M et al. *JAMA Network Open.*

eFigure 2. Symptom Frequencies Among Patients With Reported Psychiatric/Behavioural Symptoms

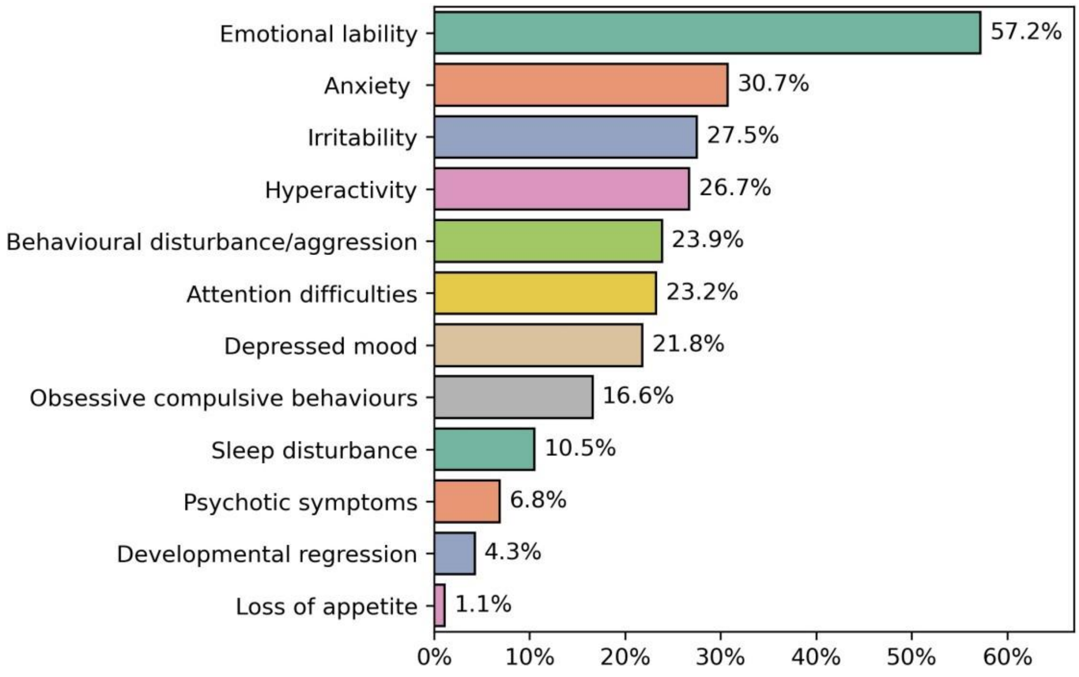

© 2024 Eyre M et al. *JAMA Network Open.*

eTable 1. Literature Search and Record Selection: Databases Searched and Numbers of Records Retrieved, Screened and Included

Database / Register	Search date	Search strategy	Records retrieved	Duplicates removed	Records screened for eligibility	Records included **
**Pubmed**	14.02.2022 (first search) 01.11.2022 (refresh of first search)	(sydenham or sydenham's or rheumatic or minor) AND chorea	**1462** (**1446** first search, **16** refresh search)	**0**	**1462**	**256**
**Embase**	14.02.2022	(sydenham* OR rheumatic OR 'minor'/exp OR minor) AND ('chorea'/exp OR chorea)	**2360**	**1668**<br>With the following automatic filter applied to original search: **NOT ([medline]/lim OR [pubmed-not-medline]/lim)**<br><br>**87**<br>Manual removal within the same source of data and further duplicates from Pubmed	**605**	**42**
**Cinhal**	18.02.2022	(MH "Sydenham Chorea") OR (Chorea AND ("Sydenham*" OR rheumatic OR minor)), Expanders – Apply equivalent subjects, Search modes – Boolean/Phrase	**178**	**127**<br>With the following automatic filter applied to original search: **Limiters – Exclude MEDLINE records**<br><br>**36**<br>Manual removal of duplicates from Pubmed, Embase	**15**	**6**
**Cochrane Library**	01.03.2022	(sydenham or sydenham's or rheumatic or minor) and chorea	**43**	**29**<br>Manual removal of duplicates from Pubmed, Embase and Cinhal	**14**	**0**

© 2024 Eyre M et al. *JAMA Network Open.*

Database / Register	Search date	Search strategy	Records retrieved	Duplicates removed	Records screened for eligibility	Records included **
**EU Clinical Trials Register, ISRCTN Registry, ClinicalTrials.gov, WHO-ICTRP**	07.03.2022	(sydenham or sydenham's or rheumatic or minor) and chorea	**10**	**3**<br>Manual removal of duplicates from Pubmed, Embase, Cinhal and Cochrane Library	**7**	**0**
**LILACS**	26.03.2022	(sydenham or sydenham's or rheumatic or minor) and chorea	**143**	**39**<br>Manual removal of duplicates from Pubmed, Embase, Cinhal, Cochrane Library and Registers	**104**	**3**

**Papers which could be retrieved for selected languages (English, Italian, Spanish, French, Portuguese) containing individualised patient data

© 2024 Eyre M et al. *JAMA Network Open.*

eTable 2. Proportions of Missing (Imputed) Data in the Multivariable Models

Predictor variable	Model for chorea duration at first episode	Model for relapsing disease course	Model for functional outcome
Age at onset	3%	9%	8%
Female	3%	5%	7%
Moderate-to-high population ARF risk	0%	0%	0%
History of other autoimmune/inflammatory diseases	37%	50%	34%
Pre-existing psychiatric/neurologic/neurodevelopmental disorders	42%	56%	38%
Evidence of preceding streptococcal infection	47%	61%	30%
Hemichorea	30%	48%	17%
Any psychiatric/behavioural symptoms	34%	55%	30%
Psychiatric symptoms prominent in initial presentation	32%	58%	35%
Impaired speech	45%	74%	39%
Worst mRS score in the acute phase	36%	63%	35%
Fever	34%	68%	37%
Carditis/valvulitis	10%	16%	7%
Arthritis/arthralgia	11%	16%	9%
Erythema marginatum/subcutaneous nodules	17%	31%	13%
Antibiotics	16%	33%	12%
Any immunotherapy given at first SC episode	0%	30%	8%
Corticosteroids	0%	30%	8%
IVIG	0%	30%	8%
Plasma exchange	0%	30%	8%
Haloperidol	0%	37%	12%
Sodium valproate	0%	37%	12%
Sodium channel blockers	0%	37%	12%
Chlorpromazine and other phenothiazines	0%	37%	12%
Benzodiazepines	0%	37%	12%
Phenobarbitone and other barbiturates	0%	37%	12%
Second-generation antipsychotics	0%	37%	12%
Antihistamines	0%	37%	12%

ARF: acute rheumatic fever; IVIG: intravenous immunoglobulin; mRS: modified Rankin Scale; SC: Sydenham's chorea.

© 2024 Eyre M et al. *JAMA Network Open.*

eTable 3. Historical Comparison of Patients With Disease Onset Before and After 1945

Variable	Disease onset 1843-1944 (n=154)	Disease onset 1945-2022 (n=1325)	p
Year of disease onset, median (IQR)	1889 (1889-1935)	2004 (1980-2013)	-
Age at disease onset (years), median (IQR)	10.0 (8.0-13.0)	10.3 (8.0-13.0)	0.259[a]
Female, n/N (%)	110/154 (71.4%)	875/1272 (68.8%)	0.503[b]
Fever during the first SC episode, n/N (%)	11/20 (55%)	66/458 (14.4%)	**<0.001**[c]
Hemichorea, n/N (%)	54/148 (36.5%)	208/664 (31.3%)	0.225[b]
Impaired speech, n/N (%)	8/16 (50%)	210/337 (62.3%)	0.322[b]
Any psychiatric/behavioural symptoms, n/N (%)	9/18 (50%)	312/484 (64.5%)	0.210[b]
Worst mRS score in the acute phase, median (IQR)	4 (3-4)	3 (3-4)	**0.042**[a]
Duration of hospitalisation (days), median (IQR)	40 (25-62.5)	21 (10-35)	**0.004**[a]
Carditis/valvulitis, n/N (%)	88/146 (60.3%)	610/1151 (53.0%)	0.097[b]
Arthritis/arthralgia, n/N (%)	48/145 (33.1%)	275/1118 (24.6%)	**0.027**[b]
Erythema marginatum/subcutaneous nodules, n/N (%)	7/98 (7%)	31/836 (3.7%)	0.107[c]
Time to chorea resolution at first episode (months), median (IQR)	2.0 (1.0-3.0)	3.0 (1.2-6.0)	**0.001**[d]
Reported relapse, n/N (%)	61/147 (41.5%)	263/766 (34.3%)	0.096[b]
Poor functional outcome, n/N (%)	5/13 (39%)	47/338 (13.9%)	**0.030**[c]

[a]Mann-Whitney U test, [b]Chi-square test, [c]Fisher's exact test, [d]Log-rank test. SC: Sydenham's chorea.

© 2024 Eyre M et al. *JAMA Network Open.*

eTable 4. Complete Descriptive Data in 1325 Patients with Sydenham's Chorea Since 1945

**DEMOGRAPHICS AND BACKGROUND HISTORY**	
Age at onset (years)	Median 10.3, mean 10.9, range 1.2-88 (d.a. 1202/1325)
Child (<18 y)	96.1% (1265/1317)
Adult	3.9% (52/1317)
Female	68.8% (875/1272)
Family history of ARF	7.6% (33/435)
History of other autoimmune/inflammatory diseases	6.0% (27/447)
Pre-existing psychiatric/neurologic/neurodevelopmental disorders	8.9% (37/418)
Pre-existing emotional hyperreactivity	3.6% (15/418)
Pre-existing anxiety or mood disorder	1.4% (6/418)
Pre-existing seizure disorder	0.7% (3/418)
Pre-existing attention deficit disorder/ADHD	0.5% (2/418)
Pre-existing autism / autism spectrum disorder	0.5% (2/418)
Country of residence / healthcare provision (only countries with >20 cases are listed)	
Brazil	24.6% (324/1317)
Turkey	19.3% (254/1317)
Italy	14.7% (194/1317)
United States	10.4% (137/1317)
United Kingdom	4.0% (53/1317)
Thailand	3.4% (45/1317)
Israel	3.3% (43/1317)
New Zealand	3.0% (40/1317)
France	2.1% (27/1317)
India	1.7% (23/1317)
Egypt	1.7% (22/1317)
Population ARF risk	
Low	85.1% (1111/1305)
Moderate-to-high	14.9% (194/1305)
**CLINICAL FEATURES OF THE FIRST SC EPISODE (WITHIN FIRST 3 MONTHS AFTER INITIAL PRESENTATION)**	
**Infection preceding SC onset**	
Symptoms of infection preceding SC onset (any)	53.4% (259/485)
Pharyngitis	33.6% (163/485)
Tonsillitis	8.5% (41/485)
Fever	3.3% (16/485)
Other	6.6% (32/485)
Unspecified	2.7% (13/485)
Weeks between infection and SC onset	Median 8, mean 10, range 0-104 (d.a. 158/259)
Antibiotics given before onset of ARF/SC	12.4% (43/347)
IM penicillin G benzathine	0.6% (2/347)
Oral penicillin	2.0% (7/347)
Oral amoxicillin	2.0% (7/347)
Other	4.0% (14/347)

© 2024 Eyre M et al. *JAMA Network Open.*

Total no. days on antibiotics at this time (single dose IM pen G = 30 days)	Median 12.5, mean 57.3, range 3-365 (d.a. 8/43)
**Initial presenting symptom of SC**	
Motor	80.2% (325/405)
Motor + Psychiatric	13.1% (53/405)
Psychiatric	6.7% (27/405)
**Motor symptoms**	
Bilateral chorea	68.7% (456/664)
Hemichorea	31.3% (208/664)
Right hemichorea	56.0% (98/175)
Left hemichorea	44.0% (77/175)
Limb involvement	99.7% (572/574)
Face involvement	75.7% (234/309)
Trunk involvement	47.4% (136/287)
Impaired mobility (any severity)	70.1% (227/324)
Impaired but able to walk without assistance (in younger patients reduction of less than 2 levels on the gross motor function scale)	36.7% (119/324)
Unable to walk without assistance (in younger patients reduction of at least 2 levels on the gross motor function scale)	21.6% (70/324)
Bedridden	11.7% (38/324)
Impaired speech (any severity)	62.3% (210/337)
Impaired fluency/articulation but mostly comprehensible (mild-moderate)	38.9% (131/337)
Able to speak but mostly incomprehensible (severe)	20.2% (68/337)
Unable to speak	3.3% (11/337)
Impaired object manipulation (any severity)	67.0% (177/264)
Impaired but able to self-care/self-feed	43.6% (115/264)
Severely impaired - fully dependent for self-care/feeding	23.5% (62/264)
Impaired chewing and swallowing (any severity)	22.7% (57/251)
Impaired (dysphagia) but able to continue oral feeding	16.3% (41/251)
Severely impaired – nasogastric tube or parenteral feeding required	6.4% (16/251)
Hypotonia	61.4% (151/246)
Motor impersistence ('milkmaid's grip')	52.8% (66/125)
Abnormal tongue movements ('darting tongue')	36.3% (53/146)
Muscle weakness	32.1% (63/196)
Pronator drift	14.7% (17/116)
Abnormal eye movements (e.g. impaired ocular pursuit)	5.7% (10/174)
**Psychiatric/behavioural symptoms**	
Any psychiatric/behavioural symptoms	64.5% (312/484)
Emotional lability	33.5% (139/415)
Anxiety	16.2% (59/364)
Irritability	14.4% (52/361)
Hyperactivity	14.0% (51/363)
Behavioural disturbance/aggression	12.7% (47/369)
Attention difficulties	12.0% (43/357)
Depressed mood	11.4% (41/360)
Obsessive compulsive behaviours	8.6% (31/359)

© 2024 Eyre M et al. *JAMA Network Open.*

Sleep disturbance	5.4% (19/353)
Psychotic symptoms (e.g. hallucinations, delusions)	3.6% (13/362)
Tics	2.5% (9/358)
Developmental regression	2.2% (8/360)
Was the patient assessed by a mental health specialist	17.6% (57/324)
Were any named assessment tools used (e.g. SDQ)	13.1% (42/321)
Was the patient assigned a formal psychiatric diagnosis (e.g. DSM/ICD-10 disorder)	9.9% (31/312)
**Other symptoms**	
Fever	14.4% (66/458)
Cognition disturbance, disorientation, confusion, memory disturbance, or deterioration of school performance	1.2% (15/1263)
Any other neurological or psychiatric symptoms reported	4.4% (56/1263)
**Severity**	
Hospital admission	90.8% (452/498)
Main indication for admission: Chorea/Neurological symptoms	93.2% (317/340)
Main indication for admission: Neurologic or psychiatric symptoms (not specified)	3.2% (11/340)
Main indication for admission: Psychiatric symptoms	1.8% (6/340)
Main indication for admission: Admission primarily for problems in another organ system (e.g. cardiac)	1.8% (6/340)
Admission duration (days)	Median 21, mean 28.3, range 2-150 (d.a. 99/452)
Worst mRS score	Median 3, mean 3.2, range 1-5 (d.a. 460/1325)
1	1.1% (5/460)
2	16.3% (75/460)
3	52.4% (241/460)
4	19.1% (88/460)
5	11.1% (51/460)
mRS source: assigned by the form completor	91.5% (421/460)
Worst UFMG Sydenham's Chorea Rating Scale (USCRS)	Median 58, mean 46.5, range 3-96 (d.a. 47/1319)
Worst Walker/Wilmshurst/Wendy Clinical Rating Scale for SC score (Walker 2012)	d.a. 0/1319
**OTHER MAJOR MANIFESTATIONS OF ACUTE RHEUMATIC FEVER**	
**Carditis**	
Was an echocardiogram ever performed?	37.6% (498/1325)
Carditis/valvulitis	53.0% (610/1151)
Clinical diagnosis (auscultatory findings) + positive echo	45.3% (140/309)
Subclinical carditis (classic auscultatory findings of valvar dysfunction either not present or not recognized by the diagnosing clinician but echo/doppler studies reveal mitral or aortic valvulitis)	34.3% (106/309)
Clinical diagnosis only (auscultatory findings), echo not done	20.4% (63/309)
Valvulitis	95.7% (336/351)
Mitral	60.4% (212/351)
Mitral + Aortic	23.1% (81/351)
Aortic	2.8% (10/351)
Other	9.4% (33/351)
Endocarditis	9.9% (29/292)
Myocarditis	1.7% (5/294)

© 2024 Eyre M et al. *JAMA Network Open.*

Pericarditis	1.7% (5/295)
Arrhythmia	1.0% (3/296)
Heart failure	2.8% (8/289)
**Joint involvement**	
Arthritis/arthralgia	24.6% (275/1118)
Polyarthritis	35.6% (63/177)
Monoarthritis	7.9% (14/177)
Polyarthralgia	48.6% (86/177)
Monoarthralgia	7.9% (14/177)
**Skin manifestations**	
Erythema marginatum/subcutaneous nodules	3.7% (31/836)
Erythema marginatum ever detected	82.8%% (24/29)
Subcutaneous nodules ever detected	17% (5/29)
**INVESTIGATION FINDINGS AT THE FIRST SC EPISODE (WITHIN FIRST 3 MONTHS AFTER INITIAL PRESENTATION)**	
**Evidence of preceding Streptococcal infection**	
Evidence of preceding streptococcal infection*	85.3% (559/655)
ASOT elevated	71.8% (393/547)
Before SC onset	3.0% (8/266)
Number of days before	Median 54.5, mean 138.8, range 7-550 (d.a. 6/8)
After SC onset	97.0% (258/266)
Number of days after	Median 14, mean 35.3, range 1-752 (d.a. 125/258)
Highest value (IU/mL)	Median 603, mean 776, range 200-3200 (d.a. 298/393)
ASOT rise on serial testing	32% (19/60)
Anti-DNAse B elevated	59.7% (80/134)
Before SC onset	0% (0/73)
Number of days before	n.a.
After SC onset	100% (73/73)
Number of days after	Median 14, mean 18.3, range 3-60 (d.a. 25/73)
Highest value (IU/mL)	Median 900, mean 1121.3, range 254-10240 (d.a. 69/80)
Anti-DNAse B rise on serial testing	21% (5/24)
Positive throat culture for GAS	37.4% (68/182)
Before SC onset	25% (16/64)
Number of days before	Median 100, mean 116.9, range 5-550 (d.a. 16/16)
After SC onset	75% (48/64)
Number of days after	Median 10, mean 22.4, range 2-90 (d.a. 25/48)
Positive rapid group A streptococcal carbohydrate antigen test	60% (3/5)
**Evidence of systemic inflammation**	
Elevated ESR	54.3% (255/470)
≥30 mm/hr	51.7% (107/209)
≥60 mm/hr	39.6% (42/106)
highest value (mm/h)	Median 30, mean 39.9, range 10-150 (d.a. 204/255)
Elevated CRP	27.6% (94/340)
highest value (mg/dL)	Median 15, mean 32.0, range 1.3-296 (d.a. 36/94)
**ECG**	
Prolonged PR interval	12.2% (41/337)

© 2024 Eyre M et al. *JAMA Network Open.*

Any other ECG abnormality	9.0% (16/177)
**Neuroimaging**	
Abnormal neuroimaging (CT and/or MRI)	16.0% (44/275)
Abnormal brain structural MRI (T1/T2/FLAIR)	19.1% (43/225)
Basal ganglia abnormal (focal swelling or T2/FLAIR hyperintensity)	7.2% (16/223)
Caudate nuclei	6.9% (14/204)
Putamina	3.4% (7/204)
Globi pallidi	0.5% (1/204)
Substantia nigra	0.0% (0/204)
White matter abnormal (focal T2/FLAIR hyperintensity)	6.7% (15/224)
Cortex abnormal (focal swelling or T2/FLAIR hyperintensity)	1.3% (3/224)
Thalamus	1.3% (3/224)
Other abnormalities	4.0% (9/224)
Abnormal brain CT	4% (3/69)
Basal ganglia abnormal (focal swelling or hypodensity)	3% (2/68)
Caudate nuclei	3% (2/69)
Putamina	1% (1/69)
Globi pallidi	0% (0/69)
White matter abnormal (focal hypodensity)	0% (0/69)
Cortex abnormal (focal swelling or hypodensity)	0% (0/69)
Any other abnormality	3% (2/69)
Abnormal brain MR angiography	11% (1/9)
Abnormal brain MR spectroscopy	67% (2/3)
Abnormal brain positron emission tomography	91% (10/11)
Abnormal brain single-photon emission computed tomography	82% (59/72)
**EEG**	
Abnormal EEG (slow/disorganized activity and/or epileptic activity)	54.9% (84/153)
Focal or diffuse slow or disorganised activity	51.7% (77/149)
Epileptic activity (epileptic discharges/electrographic seizures)	4.8% (7/147)
**CSF**	
Abnormal CSF (pleocytosis and/or positive oligoclonal bands)	25% (8/32)
Pleocytosis ≥5 cells/uL (non-bloody tap only)	13% (4/31)
Elevated CSF proteins >45 mg/dL	0% (0/30)
Oligoclonal bands positive (CSF bands unmatched in serum)	8% (2/26)
**TREATMENT OF THE FIRST SC EPISODE**	
**Antibiotics**	
Antibiotics after onset of ARF/SC	86.1% (744/867)
IM penicillin G benzathine	75.6% (591/782)
Oral penicillin	8.8% (68/770)
Oral amoxicillin	1.6% (12/770)
**Immunotherapy**	
Any immunotherapy (IT) given at first SC episode	25.7% (231/898)
Steroids (IV or oral)	23.2% (208/898)
IV or IM steroids	6.3% (56/882)
IV methylprednisolone	4.6% (40/867)
IM ACTH	1.8% (16/867)

© 2024 Eyre M et al. *JAMA Network Open.*

IV dexamethasone	0.0% (0/867)
Oral steroids	19.0% (167/881)
Oral prednisone	13.1% (111/848)
Oral deflazacort	0.4% (3/845)
Oral dexamethasone	0.1% (1/845)
Total no. weeks on steroids at 1st episode (IV + Oral)	Median 4, mean 6.9, range 0.7-52 (d.a. 136/208)
IVIG	2.3% (21/898)
Plasma exchange	1.3% (12/898)
Rituximab	0.0% (0/898)
Cyclophosphamide	0.0% (0/898)
Mycophenolate mofetil	0.0% (0/898)
Azathioprine	0.0% (0/898)
Days between SC symptom onset and first IT	Median 17, mean 38.1, range 0-730 (d.a. 135/231)
**Symptomatic pharmacological treatments**	
Any symptomatic pharmacological treatments given at first episode	78.6% (540/687)
Haloperidol	36.3% (241/663)
Valproate	20.5% (136/663)
Phenobarbital	9.4% (62/663)
Diazepam	4.5% (30/663)
Chlorpromazine	3.8% (25/663)
Carbamazepine	2.9% (19/663)
Tetrabenazine	1.4% (9/663)
Pimozide	1.1% (7/663)
Risperidone	0.9% (6/663)
Levetiracetam	0.3% (2/663)
Total weeks on symptomatic treatments at first episode	Median 8, mean 18.9, range 0.5-1040 (d.a. 169/540)
**DIAGNOSIS VALIDATION**	
Did the patient meet diagnostic criteria for ARF according to the authors	97.6% (911/930)
Revised Jones Criteria (2015)	31.0% (227/732)
New Zealand guidelines (2008)	0.1% (1/732)
WHO guidelines (2001)	3.3% (24/732)
Revised Jones Criteria (1992)	44.0% (322/732)
Other	21.6% (158/732)
**RESOLUTION OF THE FIRST SC EPISODE**	
Full resolution of chorea after the first SC episode	85.0% (529/622)
Months from initial SC onset to initial full resolution of chorea	Median 3.0, IQR 1.2-6.0, range 0.2-84 (d.a. 353/529)
**RELAPSES**	
Relapse (calculated on all patients, regardless of follow-up duration)	34.3% (263/766)
Relapses (calculated only on patients with relapse at any time OR monophasic with at least 24 months follow-up)	76.2% (263/345)
Total number of SC episodes - including first episode	Median 2, mean 2.4, range 2-9 (d.a. 240/263)
Any relapses of other ARF manifestations (without SC) as defined above	3.1% (20/637)
Any relapses of psychiatric/behavioural symptoms (without SC)	0.7% (4/564)

© 2024 Eyre M et al. *JAMA Network Open.*

Months from initial SC onset to 2nd SC episode	Median 16.0, mean 51.5, range 0.3-828 (d.a. 189/263)
Was relapse associated with ≥1 other major manifestation and/or ≥2 minor manifestations of ARF?	19.0% (27/142)
Was relapse associated with evidence of group A streptococcal infection?	32% (31/98)
Was relapse associated with any other trigger?	42.0% (55/132)
Did relapse occur while on antibiotics	52.6% (72/137)
Did relapse occur while on immunotherapy	0.5% (1/196)
Hemichorea	31% (29/95)
Any psychiatric/behavioural symptoms	32.9% (23/70)
New initiation, reinitiation or escalation of antibiotics at 2nd episode	64.6% (82/127)
New initiation, reinitiation or escalation of immunotherapy (IT) at 2nd episode	14.6% (24/164)
New initiation, reinitiation or escalation of symptomatic pharmacological treatment at 2nd episode	51.5% (67/130)
**CLINICAL COURSE AND FINAL FOLLOW-UP**	
Months from initial SC onset to final follow-up	Median 12, mean 36.6, range 0.2-912 (d.a. 720/1325)
Ongoing chorea at final follow-up	23.2% (138/595)
Any psychiatric/behavioural symptoms at final follow-up	5.9% (28/472)
Cognitive / school performance problems at final follow-up	3.0% (12/395)
Sleep disorder at final follow-up	0.5% (2/381)
Speech disorder at final follow-up	1.7% (8/462)
Was follow-up neuroimaging (>3 months after SC onset) performed	5.2% (68/1319)
Abnormal	47.1% (32/68)
Heart disease at final follow-up	12.6% (65/514)
Joint disease at final follow-up	1.2% (7/587)
Skin disease at final follow-up	0.3% (2/529)
mRS score at final follow-up (calculated on all patients with available mRS at last f-u, regardless of f-u duration)	Median 0, mean 0.4, range 0-4 (d.a. 472/1325)
mRS 0	71.0% (335/472)
mRS 1	16.9% (80/472)
mRS 2	10.2% (48/472)
mRS 3	1.5% (7/472)
mRS 4	0.4% (2/472)
mRS score at final follow-up (calculated including only patients with mRS 0-1 at any time and patients with mRS ≥2 with ≥6 months follow-up from last SC event)	Median 0, mean 0.3, range 0-3 (d.a. 203/1325)
mRS 0	75.4% (153/203)
mRS 1	16.7% (34/203)
mRS 2	6.4% (13/203)
mRS 3	1.5% (3/203)
mRS 4	0.0% (0/203)

Descriptive data are provided on patients with available information, hence the varying denominators.
*In 96 patients reported not to have evidence of preceding GAS infection, diagnostic criteria for ARF were met in 69/76 according to the authors. Other major features of ARF in these 96 patients included carditis/valvulitis in 36, arthritis/arthralgia in 22, and skin manifestations of ARF in three.

© 2024 Eyre M et al. *JAMA Network Open.*

ACTH: Adrenocorticotropic hormone; ARF: acute rheumatic fever; ASOT: antistreptolysin O titer; CRP: C-reactive protein; CSF: cerebrospinal fluid; ECG: electrocardiography; ESR: erythrocyte sedimentation rate; GAS: group A streptococcus; IM: intramuscular; IT: immunotherapy; IV: intravenous; IVIG: intravenous immunoglobulin; MRI: magnetic resonance imaging; mRS: modified Rankin Scale; SC: Sydenham's chorea.

© 2024 Eyre M et al. *JAMA Network Open.*

eTable 5. Adverse Events Associated With Immunotherapy

Treatment	Adverse events	Severe adverse events (CTCAE grades 3-5)	Detail: Severe adverse events
Plasma exchange	1/4 (25%)	1/4 (25%)	Grade 4: Septicaemia (1)
Corticosteroids	8/129 (6.2%)	1/129 (0.8%)	Grade 3: Urinary tract infection with hydronephrosis (1)
Intravenous immunoglobulin	0/16 (0%)	0/16 (0%)	

© 2024 Eyre M et al. *JAMA Network Open.*

eTable 6. Clinician-Reported Benefit from Symptomatic Medications

Medication/class	Impression of benefit	Benefit unclear	Impression of no benefit
Sodium channel blockers	18/20 (90%)	1/20 (5%)	1/20 (5%)
Antihistamines	13/16 (81%)	1/16 (6%)	2/16 (12%)
Second-generation antipsychotics	10/17 (59%)	3/17 (18%)	4/17 (24%)
Chlorpromazine and other phenothiazines	24/51 (47%)	11/51 (22%)	16/51 (31%)
Sodium valproate	51/133 (38.3%)	49/133 (36.8%)	33/133 (24.8%)
Haloperidol	91/242 (37.6%)	119/242 (49.2%)	32/242 (13.2%)
Phenobarbitone and other barbiturates	19/66 (29%)	28/66 (42%)	19/66 (29%)
Benzodiazepines	7/41 (17%)	22/41 (54%)	12/41 (29%)

© 2024 Eyre M et al. *JAMA Network Open.*

eTable 7. Adverse Events Associated With Symptomatic Medications

Medication/class	Adverse events	Severe adverse events (CTCAE grades 3-5)	Detail: Severe adverse events
Chlorpromazine and other phenothiazines	9/42 (21%)	9/42 (21%)	Grade 3: Hypertonia/Parkinsonism (9)
Second-generation antipsychotics	2/13 (15%)	0/13 (0%)	
Haloperidol	17/179 (10%)	13/179 (7%)	Grade 4: Oculogyrate crisis (1)<br>Grade 3: Hypertonia/Parkinsonism (11), psychosis and anxiety (1)
Phenobarbitone and other barbiturates	4/55 (7%)	0/55 (0%)	
Antihistamines	1/15 (7%)	0/15 (0%)	
Sodium valproate	2/87 (2%)	1/87 (1%)	Grade 3: Hypotonia and dysphagia (1)
Benzodiazepines	0/36 (0%)	0/36 (0%)	
Sodium channel blockers	0/19 (0%)	0/19 (0%)	

© 2024 Eyre M et al. *JAMA Network Open.*

eTable 8. Multivariable Model Results

	A. Association with chorea duration at first episode		B. Association with relapsing disease course		C. Association with poor functional outcome	
**Predictor variable**	**Hazard ratio* (95% CI)**	**p**	**Odds ratio (95% CI)**	**p**	**Odds ratio (95% CI)**	**p**
Age at onset <5 years	1.61 (0.71-3.67)	0.25	0.23 (0.04-1.22)	0.085	4.23 (1.17-15.27)	**0.028**
Age at onset 12-17 years	1.09 (0.78-1.53)	0.60	1.40 (0.69-2.82)	0.353	0.70 (0.27-1.81)	0.460
Age at onset ≥18 years	0.94 (0.39-2.24)	0.88	0.39 (0.09-1.78)	0.225	2.77 (0.67-11.34)	0.158
Female	0.95 (0.68-1.32)	0.75	1.36 (0.69-2.69)	0.373	0.50 (0.21-1.18)	0.113
Moderate-to-high population ARF risk	1.34 (0.89-2.02)	0.16	1.90 (0.54-6.62)	0.315	1.47 (0.54-3.99)	0.451
History of other autoimmune/inflammatory diseases	1.2 (0.47-3.08)	0.70	0.52 (0.10-2.65)	0.433	6.02 (1.31-27.57)	**0.021**
Pre-existing psychiatric/neurological/ neurodevelopmental disorders	1.55 (0.84-2.85)	0.16	4.41 (0.84-23.25)	0.080	3.06 (0.50-18.83)	0.228
Evidence of preceding streptococcal infection	0.86 (0.58-1.28)	0.45	1.05 (0.42-2.59)	0.923	2.13 (0.59-7.64)	0.247
Hemichorea	0.97 (0.69-1.38)	0.88	1.68 (0.84-3.35)	0.141	1.13 (0.50-2.58)	0.769
Any psychiatric/behavioural symptoms	0.99 (0.71-1.37)	0.93	0.65 (0.32-1.31)	0.225	1.64 (0.65-4.12)	0.294
Psychiatric symptoms prominent in initial presentation	1.09 (0.76-1.56)	0.65	0.63 (0.27-1.46)	0.280	0.91 (0.37-2.27)	0.844
Impaired speech	0.99 (0.72-1.38)	0.97	0.49 (0.24-1.01)	0.053	0.99 (0.43-2.27)	0.982
mRS score ≥4 in the acute phase	1.16 (0.84-1.61)	0.36	1.28 (0.59-2.75)	0.532	0.73 (0.30-1.77)	0.485
Fever	0.88 (0.54-1.43)	0.60	2.35 (0.36-15.18)	0.371	3.56 (0.89-14.28)	0.073
Carditis/valvulitis	0.72 (0.52-0.99)	**0.04**	1.07 (0.57-2.01)	0.830	0.24 (0.10-0.56)	**0.001**
Arthritis/arthralgia	1.07 (0.74-1.54)	0.72	3.07 (1.15-8.17)	**0.025**	1.05 (0.40-2.78)	0.920
Erythema marginatum/SC nodules	0.83 (0.47-1.47)	0.52	0.88 (0.13-5.77)	0.891	0.46 (0.06-3.81)	0.473
Antibiotics	0.76 (0.55-1.06)	0.11	0.28 (0.09-0.85)	**0.024**	2.38 (0.76-7.51)	0.138
Immunotherapy	1.51 (1.05-2.19)	**0.03**	N/A[1]	N/A[1]	N/A[1]	N/A[1]
Corticosteroids	N/A[1]	N/A[1]	0.32 (0.15-0.67)	**0.003**	0.66 (0.29-1.54)	0.338
IVIG	N/A[1]	N/A[1]	0.41 (0.10-1.64)	0.207	2.80 (0.51-15.47)	0.238
Plasma exchange	N/A[1]	N/A[1]	N/A[3]	N/A[3]	94.20 (12.51-709)	**<0.001**
Haloperidol	1.01 (0.41-2.49)	0.98	2.02 (1.01-4.03)	**0.046**	1.00 (0.44-2.28)	0.997
Sodium valproate	1.33 (0.78-2.24)	0.29	0.33 (0.15-0.71)	**0.004**	0.63 (0.23-1.78)	0.389
Sodium channel blockers	1.13 (0.56-2.27)	0.74	1.08 (0.08-14.70)	0.955	4.70 (0.40-55.39)	0.219
Chlorpromazine and other phenothiazines	1.22 (0.66-2.26)	0.52	0.66 (0.25-1.78)	0.413	0.73 (0.13-4.03)	0.717
Benzodiazepines	N/A[2]	N/A[2]	0.60 (0.18-2.01)	0.407	1.60 (0.42-6.13)	0.490
Phenobarbitone and other barbiturates	1.17 (0.4-3.43)	0.78	0.72 (0.25-2.13)	0.556	1.40 (0.44-4.46)	0.567
Second-generation antipsychotics	1.07 (0.38-2.95)	0.90	N/A[3]	N/A[3]	2.40 (0.17-33.78)	0.516
Antihistamines	N/A[2]	N/A[2]	N/A[3]	N/A[3]	N/A[3]	N/A[3]

© 2024 Eyre M et al. *JAMA Network Open.*

*Hazard ratios >1 indicate association with shorter chorea duration.
[1]Variable not included in the model: immunotherapies were included as separate variables in the logistic regression models, but as a combined (time-varying) feature in the Cox proportional hazard model.
[2] Variable not included in the model: no patients with benzodiazepines or antihistamines as monotherapy had data available on treatment start and stop times.
[3]Variable dropped from the model: if a model failed to converge due to matrix singularity, predictor variables with zero or near-zero variance in one or both outcome classes (due to insufficient numbers of patients with the feature present) were dropped until convergence was achieved.

ARF, acute rheumatic fever; IVIG, intravenous immunoglobulin; mRS, modified Rankin Scale; SC, subcutaneous.

© 2024 Eyre M et al. *JAMA Network Open.*

eTable 9. Data Missingness According to Year of Disease Onset

Predictor variable	Cases with missing data, n (%)			*p*
	**Whole dataset (N=618)**	**Onset before the year 2000 (N=309)**	**Onset since the year 2000 (N=309)**	
Impaired speech	372 (60%)	198 (64%)	174 (56%)	0.059
Fever	355 (57%)	174 (56%)	181 (59%)	0.625
Pre-existing psychiatric/neurologic/ neurodevelopmental disorders	325 (53%)	152 (49%)	173 (56%)	0.107
Psychiatric symptoms prominent in initial presentation	319 (52%)	146 (47%)	173 (56%)	**0.036**
Worst mRS score in the acute phase	310 (50%)	170 (55%)	140 (45%)	**0.020**
Evidence of preceding streptococcal infection	293 (47%)	155 (50%)	138 (45%)	0.197
History of other autoimmune/inflammatory diseases	292 (47%)	130 (42%)	162 (52%)	**0.012**
Any psychiatric/behavioural symptoms	290 (47%)	146 (47%)	144 (47%)	0.936
Hemichorea	229 (37%)	132 (43%)	97 (31%)	**0.005**
Erythema marginatum/subcutaneous nodules	145 (23%)	87 (28%)	58 (19%)	**0.008**
Antibiotics	145 (23%)	92 (30%)	53 (17%)	**<0.001**
Haloperidol	136 (22%)	91 (29%)	45 (15%)	**<0.001**
Sodium valproate	136 (22%)	91 (29%)	45 (15%)	**<0.001**
Sodium channel blockers	136 (22%)	91 (29%)	45 (15%)	**<0.001**
Chlorpromazine and other phenothiazines	136 (22%)	91 (29%)	45 (15%)	**<0.001**
Benzodiazepines	136 (22%)	91 (29%)	45 (15%)	**<0.001**
Phenobarbitone and other barbiturates	136 (22%)	91 (29%)	45 (15%)	**<0.001**
Second-generation antipsychotics	136 (22%)	91 (29%)	45 (15%)	**<0.001**
Antihistamines	136 (22%)	91 (29%)	45 (15%)	**<0.001**
Any immunotherapy given at first SC episode	107 (17%)	55 (18%)	52 (17%)	0.832
Corticosteroids	107 (17%)	55 (18%)	52 (17%)	0.832
IVIG	107 (17%)	55 (18%)	52 (17%)	0.832
Plasma exchange	107 (17%)	55 (18%)	52 (17%)	0.832
Arthritis/arthralgia	89 (14%)	42 (14%)	47 (15%)	0.647
Carditis/valvulitis	78 (13%)	40 (13%)	38 (12%)	0.904
Age at onset	48 (8%)	36 (12%)	12 (4%)	**<0.001**
Female	34 (6%)	27 (9%)	7 (2%)	**0.001**
Moderate-to-high population ARF risk	0 (0%)	0 (0%)	0 (0%)	1

© 2024 Eyre M et al. *JAMA Network Open.*

eTable 10. Nested Model for Chorea Duration at First Episode With Twentieth Century Cases Withheld

	Original model		Nested model	
**No. observations (cases)**	178 (153 events)		75 (64 events)	
**No. features (predictor variables)**	25		24*	
**Partial AIC**	1473.09		553.05	
**Partial log-likelihood**	-711.55		-252.52	
**Predictor variable**	**Hazard ratio (95% CI)**	***p***	**Hazard ratio (95% CI)**	***p***
Age at onset <5 years	1.61 (0.71-3.67)	0.25	1.25 (0.47-3.31)	0.65
Age at onset 12-17 years	1.09 (0.78-1.53)	0.60	0.99 (0.55-1.8)	0.98
Age at onset ≥18 years	0.94 (0.39-2.24)	0.88	0.59 (0.1-3.41)	0.56
Female	0.95 (0.68-1.32)	0.75	1.11 (0.65-1.88)	0.71
Moderate-to-high population ARF risk	1.34 (0.89-2.02)	0.16	1.25 (0.62-2.53)	0.54
History of other autoimmune/inflammatory diseases	1.2 (0.47-3.08)	0.70	1.28 (0.22-7.44)	0.78
Pre-existing psychiatric/neurological/ neurodevelopmental disorders	1.55 (0.84-2.85)	0.16	0.98 (0.37-2.62)	0.97
Evidence of preceding streptococcal infection	0.86 (0.58-1.28)	0.45	1.07 (0.53-2.16)	0.86
Hemichorea	0.97 (0.69-1.38)	0.88	0.66 (0.35-1.25)	0.21
Any psychiatric/behavioural symptoms	0.99 (0.71-1.37)	0.93	1.1 (0.64-1.9)	0.72
Psychiatric symptoms prominent in initial presentation	1.09 (0.76-1.56)	0.65	1.1 (0.66-1.84)	0.72
Impaired speech	0.99 (0.72-1.38)	0.97	1.01 (0.58-1.77)	0.96
mRS score ≥4 in the acute phase	1.16 (0.84-1.61)	0.36	0.87 (0.5-1.51)	0.62
Fever	0.88 (0.54-1.43)	0.60	0.99 (0.41-2.34)	0.97
Carditis/valvulitis	0.72 (0.52-0.99)	**0.04**	1.12 (0.63-1.99)	0.71
Arthritis/arthralgia	1.07 (0.74-1.54)	0.72	0.79 (0.42-1.49)	0.46
Erythema marginatum/SC nodules	0.83 (0.47-1.47)	0.52	1.11 (0.55-2.24)	0.77
Antibiotics	0.76 (0.55-1.06)	0.11	0.7 (0.24-2.02)	0.51
Immunotherapy	1.51 (1.05-2.19)	**0.03**	1.73 (0.98-3.05)	0.06
Haloperidol	1.01 (0.41-2.49)	0.98	0.87 (0.25-3)	0.82
Sodium valproate	1.33 (0.78-2.24)	0.29	0.98 (0.33-2.91)	0.97
Sodium channel blockers	1.13 (0.56-2.27)	0.74	1.42 (0.6-3.38)	0.43
Chlorpromazine and other phenothiazines	1.22 (0.66-2.26)	0.52	0.82 (0.12-5.6)	0.84
Phenobarbitone and other barbiturates	1.17 (0.4-3.43)	0.78	N/A*	N/A*
Second-generation antipsychotics	1.07 (0.38-2.95)	0.90	1.35 (0.42-4.34)	0.62

*Variable dropped from the model: if a model failed to converge due to matrix singularity, predictor variables with zero or near-zero variance in one or both outcome classes (due to insufficient numbers of patients with the feature present) were dropped until convergence was achieved.

© 2024 Eyre M et al. *JAMA Network Open.*

eTable 11. Nested Model for Relapsing Disease Course With Twentieth Century Cases Withheld

	Original model		Nested model	
**No. observations (cases)**	345		171	
**No. features (predictor variables)**	26		21*	
**Pseudo R-squared**	0.2522		0.2796	
**Log-likelihood**	-141.48		-75.071	
**Predictor variable**	**Odds ratio (95% CI)**	***p***	**Odds ratio (95% CI)**	***p***
Age at onset <5 years	0.23 (0.04-1.22)	0.085	0.44 (0.06-3.25)	0.419
Age at onset 12-17 years	1.40 (0.69-2.82)	0.353	1.84 (0.63-5.38)	0.268
Age at onset ≥18 years	0.39 (0.09-1.78)	0.225	0.53 (0.05-5.58)	0.598
Female	1.36 (0.69-2.69)	0.373	1.36 (0.53-3.50)	0.527
Moderate-to-high population ARF risk	1.90 (0.54-6.62)	0.315	0.21 (0.02-2.19)	0.191
History of other autoimmune/inflammatory diseases	0.52 (0.10-2.65)	0.433	N/A*	N/A*
Pre-existing psychiatric/neurological/ neurodevelopmental disorders	4.41 (0.84-23.25)	0.080	N/A*	N/A*
Evidence of preceding streptococcal infection	1.05 (0.42-2.59)	0.923	0.56 (0.13-2.45)	0.441
Hemichorea	1.68 (0.84-3.35)	0.141	1.48 (0.59-3.75)	0.404
Any psychiatric/behavioural symptoms	0.65 (0.32-1.31)	0.225	0.93 (0.29-2.96)	0.907
Psychiatric symptoms prominent in initial presentation	0.63 (0.27-1.46)	0.280	0.54 (0.18-1.62)	0.273
Impaired speech	0.49 (0.24-1.01)	0.053	0.37 (0.13-1.06)	0.065
mRS score ≥4 in the acute phase	1.28 (0.59-2.75)	0.532	3.22 (0.85-12.25)	0.086
Fever	2.35 (0.36-15.18)	0.371	4.04 (0.14-115.34)	0.414
Carditis/valvulitis	1.07 (0.57-2.01)	0.830	0.68 (0.28-1.66)	0.397
Arthritis/arthralgia	3.07 (1.15-8.17)	**0.025**	3.50 (0.82-14.88)	0.090
Erythema marginatum/SC nodules	0.88 (0.13-5.77)	0.891	N/A*	N/A*
Antibiotics	0.28 (0.09-0.85)	**0.024**	N/A*	N/A*
Corticosteroids	0.32 (0.15-0.67)	**0.003**	0.21 (0.08-0.55)	**0.001**
IVIG	0.41 (0.10-1.64)	0.207	0.41 (0.09-1.79)	0.236
Haloperidol	2.02 (1.01-4.03)	**0.046**	1.87 (0.74-4.73)	0.187
Sodium valproate	0.33 (0.15-0.71)	**0.004**	0.30 (0.10-0.89)	**0.029**
Sodium channel blockers	1.08 (0.08-14.70)	0.955	1.91 (0.12-30.39)	0.647
Chlorpromazine and other phenothiazines	0.66 (0.25-1.78)	0.413	0.21 (0.04-1.24)	0.085
Benzodiazepines	0.60 (0.18-2.01)	0.407	0.87 (0.07-10.86)	0.917
Phenobarbitone and other barbiturates	0.72 (0.25-2.13)	0.556	N/A*	N/A*

*Variable dropped from the model: if a model failed to converge due to matrix singularity, predictor variables with zero or near-zero variance in one or both outcome classes (due to insufficient numbers of patients with the feature present) were dropped until convergence was achieved. It was not possible to include antibiotics in the nested model due to insufficient numbers of patients not given antibiotics in the last two decades (**Figure 1**).

© 2024 Eyre M et al. *JAMA Network Open.*

eTable 12. Nested Model for Poor Functional Outcome With Twentieth Century Cases Withheld

	Original model		Nested model	
**No. observations (cases)**	338		179	
**No. features (predictor variables)**	28		23*	
**Pseudo R-squared**	0.2154		0.2600	
**Log-likelihood**	-106.94		-57.444	
**Predictor variable**	**Odds ratio (95% CI)**	***p***	**Odds ratio (95% CI)**	***p***
Age at onset <5 years	4.23 (1.17-15.27)	**0.028**	2.01 (0.35-11.46)	0.430
Age at onset 12-17 years	0.70 (0.27-1.81)	0.460	0.49 (0.13-1.90)	0.304
Age at onset ≥18 years	2.77 (0.67-11.34)	0.158	1.29 (0.18-9.07)	0.797
Female	0.50 (0.21-1.18)	0.113	0.83 (0.24-2.84)	0.766
Moderate-to-high population ARF risk	1.47 (0.54-3.99)	0.451	2.47 (0.60-10.17)	0.209
History of other autoimmune/inflammatory diseases	6.02 (1.31-27.57)	**0.021**	N/A*	N/A*
Pre-existing psychiatric/neurological/ neurodevelopmental disorders	3.06 (0.50-18.83)	0.228	N/A*	N/A*
Evidence of preceding streptococcal infection	2.13 (0.59-7.64)	0.247	0.18 (0.04-0.86)	**0.031**
Hemichorea	1.13 (0.50-2.58)	0.769	1.96 (0.67-5.77)	0.220
Any psychiatric/behavioural symptoms	1.64 (0.65-4.12)	0.294	4.59 (1.05-20.04)	**0.043**
Psychiatric symptoms prominent in initial presentation	0.91 (0.37-2.27)	0.844	1.86 (0.57-6.07)	0.303
Impaired speech	0.99 (0.43-2.27)	0.982	0.47 (0.16-1.41)	0.179
mRS score ≥4 in the acute phase	0.73 (0.30-1.77)	0.485	1.23 (0.38-4.04)	0.730
Fever	3.56 (0.89-14.28)	0.073	1.36 (0.15-12.70)	0.787
Carditis/valvulitis	0.24 (0.10-0.56)	**0.001**	0.17 (0.05-0.53)	**0.002**
Arthritis/arthralgia	1.05 (0.40-2.78)	0.920	1.88 (0.49-7.29)	0.359
Erythema marginatum/SC nodules	0.46 (0.06-3.81)	0.473	0.69 (0.05-9.66)	0.785
Antibiotics	2.38 (0.76-7.51)	0.138	N/A*	N/A*
Corticosteroids	0.66 (0.29-1.54)	0.338	1.26 (0.42-3.81)	0.684
IVIG	2.80 (0.51-15.47)	0.238	2.09 (0.33-13.08)	0.432
Plasma exchange	94.20 (12.51-709)	**<0.001**	N/A*	N/A*
Haloperidol	1.00 (0.44-2.28)	0.997	1.76 (0.58-5.37)	0.319
Sodium valproate	0.63 (0.23-1.78)	0.389	0.54 (0.15-1.88)	0.331
Sodium channel blockers	4.70 (0.40-55.39)	0.219	2.17 (0.14-33.62)	0.579
Chlorpromazine and other phenothiazines	0.73 (0.13-4.03)	0.717	0.25 (0.01-9.09)	0.452
Benzodiazepines	1.60 (0.42-6.13)	0.490	0.31 (0.02-4.30)	0.379
Phenobarbitone and other barbiturates	1.40 (0.44-4.46)	0.567	10.58 (1.43-78.00)	**0.021**
Second-generation antipsychotics	2.40 (0.17-33.78)	0.516	N/A*	N/A*

*Variable dropped from the model: if a model failed to converge due to matrix singularity, predictor variables with zero or near-zero variance in one or both outcome classes (due to insufficient numbers of patients with the feature present) were dropped until convergence was achieved.

© 2024 Eyre M et al. *JAMA Network Open.*

eTable 13. Outcome Distributions for Variables With High Missingness in the Chorea Duration at First Episode Model

Predictor variable	Missing	Chorea duration at first episode (months), median (IQR)				*p* (missing vs. complete)
		Predictor data missing	Predictor data complete	Predictor data complete		
				Predictor present	Predictor absent	
History of other autoimmune/inflammatory diseases	37%	1.0 (0.6-2.0)	1.8 (1.2-4.7)	1.5 (1.3-1.8)	1.8 (1.2-4.7)	<0.001
Pre-existing psychiatric/neurologic /neurodevelopmental disorders	42%	1.1 (0.7-2.3)	1.7 (1.2-4.7)	1.5 (0.2-24.0)	1.8 (1.2-4.0)	0.009
Hemichorea	30%	1.0 (0.6-1.3)	1.8 (1.2-5.0)	2.0 (1.0-17.0)	1.8 (1.2-4.0)	<0.001
Psychiatric symptoms prominent in initial presentation	32%	1.0 (0.6-2.0)	1.6 (1.1-4.7)	1.2 (1.0-2.9)	1.8 (1.2-5.0)	0.001

© 2024 Eyre M et al. *JAMA Network Open.*

eTable 14. Outcome Distributions for Variables With High Missingness in the Relapsing Disease Course Model

Predictor variable	Missing	Proportion with relapsing course, n/N (%)				*p* (missing vs. complete)
		Predictor data missing	Predictor data complete	Predictor data complete		
				Predictor present	Predictor absent	
History of other autoimmune/inflammatory diseases	50%	148/174 (85%)	115/171 (67%)	5/8 (62%)	110/163 (67%)	<0.001
Pre-existing psychiatric/neurologic /neurodevelopmental disorders	56%	161/193 (83%)	102/152 (67%)	10/11 (91%)	92/141 (65%)	0.001
Evidence of preceding streptococcal infection	61%	182/209 (87%)	81/136 (60%)	60/107 (56%)	21/29 (72%)	<0.001
Hemichorea	48%	142/165 (86%)	121/180 (67%)	49/61 (80%)	72/119 (61%)	<0.001
Any psychiatric/behavioural symptoms	55%	167/191 (87%)	96/154 (62%)	47/92 (51%)	49/62 (79%)	<0.001
Psychiatric symptoms prominent in initial presentation	58%	172/200 (86%)	91/145 (63%)	9/24 (38%)	82/121 (68%)	<0.001
Impaired speech	74%	219/254 (86%)	44/91 (48%)	20/36 (56%)	17/32 (53%)	<0.001
Worst mRS score in the acute phase	63%	186/216 (86%)	77/129 (60%)	mRS=1: 2/2 (100%)<br>mRS=2: 14/20 (70%)<br>mRS=3: 45/68 (66%)<br>mRS=4: 10/18 (56%)<br>mRS=5: 6/21 (29%)		<0.001
Fever	68%	202/233 (87%)	61/112 (54%)	7/9 (78%)	54/103 (52%)	<0.001
Antibiotics	33%	97/113 (86%)	166/232 (72%)	124/187 (66%)	42/45 (93%)	0.003

© 2024 Eyre M et al. *JAMA Network Open.*

Any immunotherapy given at first SC episode	30%	96/102 (94%)	167/243 (69%)	28/67 (42%)	139/176 (79%)	<0.001
Corticosteroids	30%	96/102 (94%)	167/243 (69%)	22/59 (37%)	145/184 (79%)	<0.001
IVIG	30%	96/102 (94%)	167/243 (69%)	5/10 (50%)	162/233 (70%)	<0.001
Plasma exchange	30%	96/102 (94%)	167/243 (69%)	2/2 (100%)	165/241 (68%)	<0.001
Haloperidol	37%	116/127 (91%)	147/218 (67%)	59/75 (79%)	88/143 (62%)	<0.001
Sodium valproate	37%	116/127 (91%)	147/218 (67%)	18/42 (43%)	129/176 (73%)	<0.001
Sodium channel blockers	37%	116/127 (91%)	147/218 (67%)	3/3 (100%)	144/215 (67%)	<0.001
Chlorpromazine and other phenothiazines	37%	116/127 (91%)	147/218 (67%)	5/16 (31%)	142/202 (70%)	<0.001
Benzodiazepines	37%	116/127 (91%)	147/218 (67%)	6/12 (50%)	141/206 (68%)	<0.001
Phenobarbitone and other barbiturates	37%	116/127 (91%)	147/218 (67%)	15/23 (65%)	132/195 (68%)	<0.001
Second-generation antipsychotics	37%	116/127 (91%)	147/218 (67%)	0/1 (0%)	147/217 (68%)	<0.001
Antihistamines	37%	116/127 (91%)	147/218 (67%)	7/7 (100%)	140/211 (66%)	<0.001

© 2024 Eyre M et al. *JAMA Network Open.*

eTable 15. Outcome Distributions for Variables With High Missingness in the Poor Functional Outcome Model

Predictor variable	Missing	Proportion with poor functional outcome, n/N (%)				*p* (missing vs. complete)
		Predictor data missing	Predictor data complete	Predictor data complete		
				Predictor present	Predictor absent	
History of other autoimmune/inflammatory diseases	34%	24/114 (21%)	23/224 (10%)	4/12 (33%)	19/212 (9%)	0.012
Pre-existing psychiatric/neurologic /neurodevelopmental disorders	38%	29/128 (23%)	18/210 (9%)	2/10 (20%)	16/200 (8%)	0.001
Evidence of preceding streptococcal infection	30%	21/100 (21%)	26/238 (11%)	23/199 (12%)	3/39 (8%)	0.024
Impaired speech	39%	27/131 (21%)	20/207 (10%)	7/75 (9%)	8/68 (12%)	0.006
Fever	37%	25/126 (20%)	22/212 (10%)	3/16 (19%)	19/196 (10%)	0.022

© 2024 Eyre M et al. *JAMA Network Open.*

eTable 16. Nested Model for Chorea Duration at First Episode With Cases Missing Data in ≥1 Missing Not at Random (MNAR) Variables Withheld

	Original model		Nested model	
**No. observations (cases)**	178 (153 events)		100 (85 events)	
**No. features (predictor variables)**	25		25	
**Partial AIC**	1473.09		720.50	
**Partial log-likelihood**	-711.55		-335.25	
**Predictor variable**	**Hazard ratio (95% CI)**	***p***	**Hazard ratio (95% CI)**	***p***
Age at onset <5 years	1.61 (0.71-3.67)	0.25	2.19 (0.47-10.26)	0.32
Age at onset 12-17 years	1.09 (0.78-1.53)	0.60	1.27 (0.78-2.06)	0.34
Age at onset ≥18 years	0.94 (0.39-2.24)	0.88	1 (0.34-2.93)	1
Female	0.95 (0.68-1.32)	0.75	0.89 (0.57-1.39)	0.61
Moderate-to-high population ARF risk	1.34 (0.89-2.02)	0.16	1.13 (0.57-2.26)	0.72
History of other autoimmune/inflammatory diseases	1.2 (0.47-3.08)	0.70	1.7 (0.58-4.98)	0.34
Pre-existing psychiatric/neurological/ neurodevelopmental disorders	1.55 (0.84-2.85)	0.16	1.09 (0.34-3.47)	0.88
Evidence of preceding streptococcal infection	0.86 (0.58-1.28)	0.45	0.93 (0.49-1.77)	0.83
Hemichorea	0.97 (0.69-1.38)	0.88	0.73 (0.45-1.18)	0.19
Any psychiatric/behavioural symptoms	0.99 (0.71-1.37)	0.93	0.95 (0.6-1.5)	0.82
Psychiatric symptoms prominent in initial presentation	1.09 (0.76-1.56)	0.65	1.02 (0.55-1.88)	0.96
Impaired speech	0.99 (0.72-1.38)	0.97	0.82 (0.49-1.36)	0.44
mRS score ≥4 in the acute phase	1.16 (0.84-1.61)	0.36	1.07 (0.68-1.68)	0.78
Fever	0.88 (0.54-1.43)	0.60	0.6 (0.22-1.59)	0.3
Carditis/valvulitis	0.72 (0.52-0.99)	**0.04**	0.7 (0.45-1.08)	0.11
Arthritis/arthralgia	1.07 (0.74-1.54)	0.72	1.16 (0.68-1.97)	0.6
Erythema marginatum/SC nodules	0.83 (0.47-1.47)	0.52	0.77 (0.22-2.72)	0.68
Antibiotics	0.76 (0.55-1.06)	0.11	0.9 (0.57-1.42)	0.64
Immunotherapy	1.51 (1.05-2.19)	**0.03**	1.77 (1.06-2.95)	**0.03**
Haloperidol	1.01 (0.41-2.49)	0.98	0.8 (0.16-3.91)	0.78
Sodium valproate	1.33 (0.78-2.24)	0.29	1.02 (0.39-2.68)	0.97
Sodium channel blockers	1.13 (0.56-2.27)	0.74	1.54 (0.45-5.24)	0.49
Chlorpromazine and other phenothiazines	1.22 (0.66-2.26)	0.52	1.21 (0.59-2.45)	0.6
Phenobarbitone and other barbiturates	1.17 (0.4-3.43)	0.78	1.16 (0.37-3.67)	0.79
Second-generation antipsychotics	1.07 (0.38-2.95)	0.90	0.5 (0.08-3.26)	0.47

© 2024 Eyre M et al. *JAMA Network Open.*

eTable 17. Nested Model for Poor Functional Outcome With Cases Missing Data in ≥1 Missing Not at Random (MNAR) Variables Withheld

	Original model		Nested model	
**No. observations (cases)**	338		116	
**No. features (predictor variables)**	28		20*	
**Pseudo R-squared**	0.2154		0.3295	
**Log-likelihood**	-106.94		-21.220	
**Predictor variable**	**Odds ratio (95% CI)**	***p***	**Odds ratio (95% CI)**	***p***
Age at onset <5 years	4.23 (1.17-15.27)	**0.028**	N/A*	N/A*
Age at onset 12-17 years	0.70 (0.27-1.81)	0.460	0.62 (0.05-7.51)	0.705
Age at onset ≥18 years	2.77 (0.67-11.34)	0.158	23.88 (0.31-1814.50)	0.151
Female	0.50 (0.21-1.18)	0.113	2.60 (0.26-26.30)	0.418
Moderate-to-high population ARF risk	1.47 (0.54-3.99)	0.451	1.57 (0.09-26.50)	0.754
History of other autoimmune/inflammatory diseases	6.02 (1.31-27.57)	**0.021**	3.23 (0.05-190.89)	0.573
Pre-existing psychiatric/neurological/ neurodevelopmental disorders	3.06 (0.50-18.83)	0.228	0.93 (0.01-79.16)	0.973
Evidence of preceding streptococcal infection	2.13 (0.59-7.64)	0.247	1.24 (0.12-12.87)	0.859
Hemichorea	1.13 (0.50-2.58)	0.769	0.18 (0.01-2.87)	0.222
Any psychiatric/behavioural symptoms	1.64 (0.65-4.12)	0.294	7.34 (0.55-98.69)	0.133
Psychiatric symptoms prominent in initial presentation	0.91 (0.37-2.27)	0.844	0.26 (0.01-5.14)	0.379
Impaired speech	0.99 (0.43-2.27)	0.982	1.08 (0.12-9.73)	0.946
mRS score ≥4 in the acute phase	0.73 (0.30-1.77)	0.485	0.57 (0.06-5.51)	0.629
Fever	3.56 (0.89-14.28)	0.073	N/A*	N/A*
Carditis/valvulitis	0.24 (0.10-0.56)	**0.001**	0.14 (0.01-1.66)	0.118
Arthritis/arthralgia	1.05 (0.40-2.78)	0.920	0.06 (0.00-10.64)	0.293
Erythema marginatum/SC nodules	0.46 (0.06-3.81)	0.473	N/A*	N/A*
Antibiotics	2.38 (0.76-7.51)	0.138	N/A*	N/A*
Corticosteroids	0.66 (0.29-1.54)	0.338	0.08 (0.00-3.18)	0.177
IVIG	2.80 (0.51-15.47)	0.238	N/A*	N/A*
Plasma exchange	94.20 (12.51-709)	**<0.001**	N/A*	N/A*
Haloperidol	1.00 (0.44-2.28)	0.997	1.85 (0.21-16.39)	0.579
Sodium valproate	0.63 (0.23-1.78)	0.389	N/A*	N/A*
Sodium channel blockers	4.70 (0.40-55.39)	0.219	0.22 (0.01-4.62)	0.330
Chlorpromazine and other phenothiazines	0.73 (0.13-4.03)	0.717	0.97 (0.01-80.39)	0.991
Benzodiazepines	1.60 (0.42-6.13)	0.490	1.54 (0.03-76.48)	0.828
Phenobarbitone and other barbiturates	1.40 (0.44-4.46)	0.567	1.13 (0.06-20.72)	0.937
Second-generation antipsychotics	2.40 (0.17-33.78)	0.516	N/A*	N/A*
Antihistamines	N/A*	N/A*	N/A*	N/A*

*Variable dropped from the model: if a model failed to converge due to matrix singularity, predictor variables with zero or near-zero variance in one or both outcome classes (due to insufficient numbers of patients with the feature present) were dropped until convergence was achieved.

© 2024 Eyre M et al. *JAMA Network Open.*
