# Supplementary material for: Treatments and Outcomes Among Patients with Sydenham Chorea: A Meta-Analysis
Source: JAMA Netw Open. 2024 Apr 16;7(4):e246792. doi: 10.1001/jamanetworkopen.2024.6792 (PMC11022117; doi:10.1001/jamanetworkopen.2024.6792)
Supplement: Supplement 2. — Nonauthor Collaborators. Members of the Sydenham’s Chorea Systematic Literature Review Working Group [file jamanetwopen-e246792-s002.pdf]

Supplemental Online Content: Nonauthor Collaborators
*First name, last name, and suffix (if applicable) are required and will appear in PubMed.

*Group Name(s): Sydenham's Chorea Systematic Literature Review Working Group						
***First Name and Middle Initial(s)**	***Last Name**	***Suffix (eg, Jr, III)**	Academic Degrees	Institution	Location (city, state/province, country)	Role or Contribu eg, chair, princip investigator
Pakinam A	Aty-Marzouk		MD	Department of Pediatric Cardiology, Faculty of Medicine, Cairo University Children Hospitals	Cairo, Egypt	Data collection
Francisco	Cardoso		MD, PhD	Movement Disorders Unit, Neurology Service, Internal Medicine Department, Federal University of Minas Gerais	Minas Gerais, Brazil	Data collection
André	Cavalcanti		MD, PhD	Pediatric Rheumatology Unit, Clinical Hospital of the Federal University of Pernambuco	Recife, Brazil	Data collection
Yvonne	Galloway		BSc	Institute of Environmental Science and Research	Wellington, New Zealand	Data collection
Susan	Jack		MD, PhD	1. Te Whatu Ora, National Public Health Service - Southern; 2. University of Otago	Dunedin, New Zealand	Data collection
Debora	Maia		MD	1. Hospital das Clínicas, Federal University of Minas Gerais; 2. Movement Disorders Unit, Neurology Service, Internal Medicine Department, Federal University of Minas Gerais	Minas Gerais, Brazil	Data collection
Achille	Marino		MD, PhD	Pediatric Rheumatology, ASST G.Pini-CTO	Milan, Italy	Data collection
Alessandro	Orsini		MD	Pediatric Neurology, Pediatric University Department, Azienda Ospedaliera Universitaria Pisana, University of Pisa	Pisa, Italy	Data collection
Stefano	Sartori		MD, PhD	1. Paediatric Neurology and Neurophysiology Unit, Department of Women's and Children's Health, University Hospital of Padova; 2. Neuroimmunology Group, Paediatric Research Institute "Città della Speranza"	Padova, Italy	Data collection
