# Supplementary material for: Treatments and Outcomes Among Patients with Sydenham Chorea: A Meta-Analysis
Source: JAMA Netw Open. 2024 Apr 16;7(4):e246792. doi: 10.1001/jamanetworkopen.2024.6792 (PMC11022117; doi:10.1001/jamanetworkopen.2024.6792)
Supplement: Supplement 3. — Data Sharing Statement [file jamanetwopen-e246792-s003.pdf]

Data Sharing Statement

Eyre. Treatments and Outcomes Among Patients with Sydenham Chorea. *JAMA Netw Open*. Published April 16, 2024. doi:10.1001/jamanetworkopen.2024.6792

Data
Data available: No
